# Engineering Nanohole-Etched Quantum Dots for Telecom-Band Single-Photon Generation

**DOI:** 10.1021/acsnano.5c17982

**Published:** 2026-01-09

**Authors:** Ian M. Masson, Aden Hageman, Caleb Whittier, David Montealegre, Bhaveshkumar Kamaliya, Nabil D. Bassim, John P. Prineas, Ravitej Uppu

**Affiliations:** † Department of Physics and Astronomy, 681072 The University of Iowa, Iowa City, Iowa 52242, United States; ‡ Department of Materials Science and Engineering, 3710 McMaster University, Hamilton, Ontario L8S 4L7, Canada; ¶ Canadian Centre for Electron Microscopy, 3710 McMaster University, Hamilton, Ontario L8S 4M1, Canada

**Keywords:** quantum dots, single-photon sources, quantum communication, epitaxy, telecom wavelength

## Abstract

Bright and high-purity single-photon sources at telecom wavelengths are essential for scalable quantum networks. Nanohole-etched GaSb/AlGaSb quantum dots (QDs) are an emerging platform for telecom-band emitters, offering freedom from strain-induced decoherence and indium-related nuclear spin noise of conventional InGaAs QDs. Here, we present a comprehensive optical spectroscopy study that reveals correlations between nanohole morphology, exciton recombination dynamics, and single-photon performance in GaSb QDs. Shallow nanoholes lead to ultrafast charge transfer that limits optical coherence, whereas deeper nanoholes yield clean neutral-exciton emission with a high bright-to-dark state branching ratio (98 ± 1%), indicating favorable conditions for efficient photon generation. Under pulsed quasi-resonant excitation, these QDs exhibit significantly enhanced single-photon purity with *g* ^(2)^(0) = 0.029 ± 0.011, compared to above-band excitation (*g* ^(2)^(0) = 0.18 ± 0.05). Polarization-resolved measurements across tens of QDs further reveal ultrasmall fine-structure splitting of the neutral exciton (11 ± 5 μeV), relevant for entangled-photon generation at telecom wavelengths. These results highlight the potential of GaSb QDs for high-performance quantum emitters and scalable spin-photon interfaces in the telecom band.

## Graphical abstract

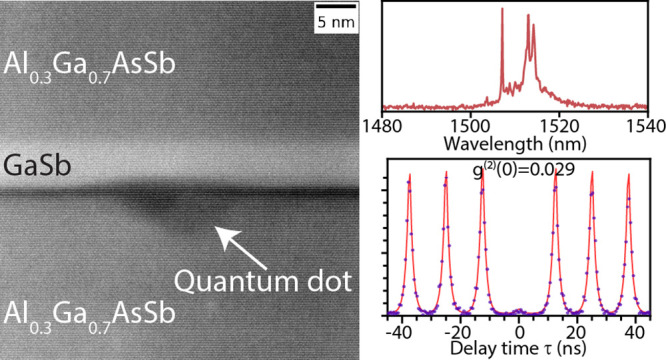

## Introduction

Implementing large-scale quantum networks for secure quantum communication and distributed quantum computing requires efficient quantum emitters capable of generating single and entangled photons.^1–3^ Specifically, emitters that support spin-photon interfaces are essential for realizing quantum repeaters that ensure loss-tolerant quantum information transfer.^4,5^ Epitaxial semiconductor quantum dots (QDs) have emerged as a leading platform for coherent single-photon generation due to their short radiative lifetimes, near-unity internal quantum efficiency, and transform-limited line widths.^6,7^ Furthermore, the availability of advanced nanofabrication processes supports the integration of QDs within scalable photonic circuitry for photon extraction and quantum information processing.^3,8,9^ These attributes have enabled bright single- and entangled-photon sources.^10–12^ Moreover, controlled loading of single charge carriers in QDs has enabled spin-photon entanglement,^13–15^ a key resource for generating multiphoton entanglement relevant for quantum repeaters.^16–18^

Despite these advantages, state-of-the-art photon sources employ InGaAs/GaAs QDs grown via strain-based methods, emitting around 930 nm—a suboptimal wavelength for quantum networking due to high optical losses in fibers and silicon photonic circuits.^19–21^ While quantum frequency conversion has succeeded in shifting the QD emission to telecom wavelengths using difference frequency generation,^22–25^ practical challenges in mitigating the intensity and frequency noise of the pump laser limit the source efficiency and photon indistinguishability.^26,27^ A more direct solution is to develop QDs emitting in the telecom band (1300–1600 nm),^28^ achieved either through strain engineering InGaAs/GaAs QDs^29–31^ or leveraging substrates with lower lattice mismatch (e.g., InAs/InP QDs).^32–34^ However, residual strain and nuclear-spin-induced decoherence from spin-9/2 indium nuclei significantly limit spin coherence times, posing challenges for scalable spin-photon interfaces.^35,36^

Nanohole-etching-based QD growth presents an exciting alternative to circumvent these limitations.^37^ This approach utilizes group-III (e.g., Al, Ga, In) atom droplets to etch nanohole templates into substrates, enabling strain-free QD nucleation.^38–41^ Successfully implemented for GaAs/AlGaAs QDs emitting around 780 nm, this technique has yielded highly symmetric QDs with spin coherence times exceeding 100 μs.^42,43^ Inspired by these advancements, GaSb/AlGaSb QDs have recently been proposed as an indium-free, telecom-band alternative.^44–46^ Initial studies of QD ensembles indicate promising optical properties, including emission around 1480 nm and favorable exciton dynamics.^47^ However, a key challenge remains: the impact of nanohole morphology on exciton recombination, charge-transfer dynamics, and photon purity has not been systematically investigated experimentally. Addressing this knowledge gap is essential for optimizing the efficiency and photon quality of these telecom-band QDs, a critical step toward scalable spin-photon interfaces.

In this work, we perform detailed spectroscopy of single GaSb/AlGaSb QDs and identify correlations between nanohole morphology, exciton recombination dynamics, and single-photon emission characteristics. We demonstrate bright single-photon emission in the telecom band with high photon purity (*g* ^(2)^(0) = 0.029). By elucidating structure–property relationships in nanohole-etched GaSb/AlGaSb QDs, these results provide insights relevant to the growth and optimization of telecom-band quantum light sources and spin-photon interfaces for scalable quantum networks.

## Results and Discussion

### Nanohole Etching and QD Growth

GaSb QDs are grown using the local droplet etching technique in a solid-source molecular beam epitaxy system (Veeco Gen20) equipped with valved arsenic (As) and antimony (Sb) cracker sources. The layer structure of the samples is illustrated in Figure 1a. The process begins with oxide removal from epi-ready GaSb(001) wafers by heating to 535 °C (pyrometer) under ultrahigh vacuum. A 110 nm GaSb buffer layer is deposited, followed by the etching of nanoholes into a lattice-matched 110 nm Al_0.3_Ga_0.7_AsSb layer using aluminum (Al) droplets. The Al_0.3_Ga_0.7_AsSb layer thickness was chosen to support a half-wave vertical cavity when combined with distributed Bragg reflectors in future device structures. Al is deposited at 395 °C under a significantly reduced Sb flux, 100-fold lower than required for GaSb layer-by-layer growth. The deposited Al self-assembles into droplets via Volmer–Weber growth, minimizing surface and interface energies. Al droplets locally dissolve the Al_0.3_Ga_0.7_AsSb layer, and etching is driven by the diffusion of layer atoms into the droplet. Subsequent annealing under low Sb-flux results in etched nanoholes, which agrees with previous reports for AlGaAs.^48,49^

**Figure 1:**
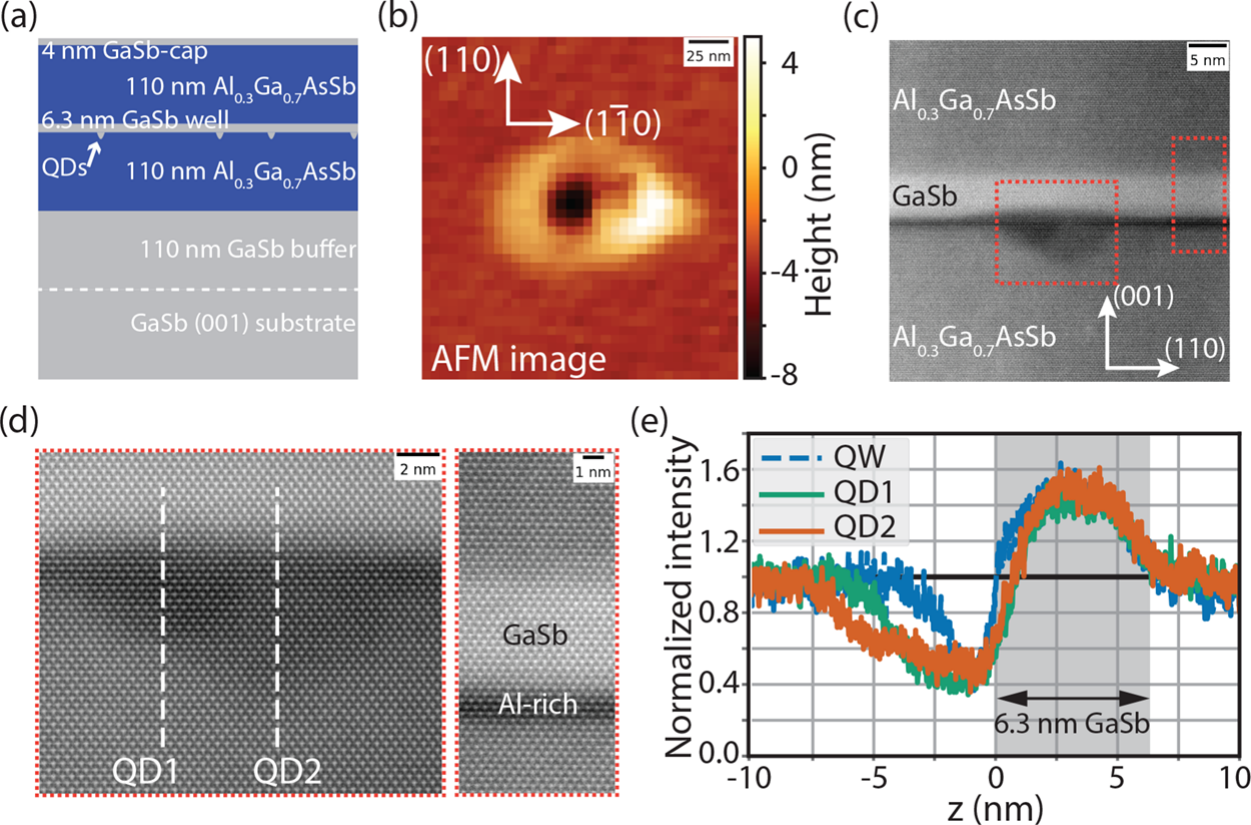
(a) Cross-section illustrating the sample layers along the growth direction. Quantum dots (QDs) were formed by infilling nanoholes in the Al_0.3_Ga_0.7_AsSb layer using aluminum local droplet etching. (b) Atomic force microscopy (AFM) image of a nanohole, revealing an 8 nm dip surrounded by a 2.5 nm tall ring. (c) Scanning transmission electron microscopy (STEM) image of a nanohole infilled with GaSb shows qualitative compositional variations at and away from the QD. (d) Higher resolution images of regions identified with red dotted boxes in (c) reveal a few monolayers of an aluminum-rich layer at the nanohole sidewalls. Two QDs infilling nanoholes of different depths are observed in the sample cross-section. (e) Scattered intensity, normalized to the signal from the Al_0.3_Ga_0.7_AsSb region, along vertical slices marked by dashed white lines in (d). The gray-shaded region marks the quantum well (QW) formed by the 6.3 nm GaSb layer.

Two samples with different Al coverages were grown to control the nanohole size and density. *Sample A*, with a high Al coverage (3.2 monolayers), exhibited a higher nanohole density with a bimodal distribution (average relief 11 ± 2 nm, areal density ∼ 0.2 μm^–2^) together with a large number of much shallower depressions (<4 nm, density >10 μm^–2^).^44,50^ *Sample B*, grown at a slightly elevated temperature (410 °C) with lower Al coverage (2.75 monolayers), produced a lower density of nearly uniform-sized nanoholes (average relief 22 ± 3 nm, density ∼ 0.04 μm^–2^) without a shallow population. Atomic force microscopy (AFM) analysis of *Sample A* reveals nanoholes with a depth of 8.0 nm, surrounded by a 2.5 nm tall nanoring of 48 nm diameter (Figure 1b). QDs are formed by infilling the nanoholes by depositing a 6.3 nm GaSb layer. Subsequently, a 110 nm Al_0.3_Ga_0.7_AsSb barrier layer was deposited, followed by a 4 nm GaSb capping layer to prevent oxidation. Further details of the AFM characterization, including representative images and statistics, are provided in the Supporting Information.

Building on the AFM analysis of nanohole morphology, the structural properties of QDs in *Sample A* were investigated using scanning transmission electron microscopy (STEM). A cross-sectional high-angle annular dark-field (HAADF) STEM image of an infilled nanohole QD is shown in Figure 1c, with additional images of multiple QDs provided in the Supporting Information. Regions with higher Al concentrations appear darker in HAADF-STEM images due to reduced electron scattering at high angles from the lower atomic number Al atoms. Two distinct Al-enriched features are evident. First, a planar Al-rich interfacial layer of ∼3 monolayers is observed away from the nanoholes at the GaSb/Al_0.3_Ga_0.7_AsSb interface (Figure 1d). This interfacial layer corresponds to the critical Al coverage required to saturate the group-V–terminated surface before droplet nucleation, consistent with the ∼2 ML value reported earlier.^47^ The slightly larger thickness observed in the STEM image could be attributed to interdiffusion during subsequent growth. Second, within the etched nanohole regions we observe extended darker contrast even after infilling with GaSb. We interpret this as local Al enrichment of the nanohole sidewalls during droplet etching, consistent with the lower volatility and slower desorption kinetics of Al compared to Ga observed in AlGaAs-based droplet-etching systems.^51^

Analysis of the QD profiles reveals (112)-type sidewall faceting with a facet angle of 35 ± 2°, consistent with AFM-measured nanohole profiles along the (110) direction. While equilibrium nanohole shapes are expected to feature (111)-type facets, previous studies on AlGaAs have associated lower growth temperatures with (11*n*)-facets due to the faster etch rates for larger *n*.^52^ Higher magnification HAADF-STEM images (Figure 1d) are consistent with lattice-matched growth. Moreover, it reveals two laterally overlapping QDs within the sample cross-section, consistent with the high nanohole density and bimodal distribution of nanoholes observed in AFM analysis of *Sample A*. The HAADF intensity profiles (Figure 1e), normalized to the signal from the Al_0.3_Ga_0.7_AsSb layer and Fourier-filtered to remove the periodic lattice fringes, corroborate these observations. We select *z* = 0 as the top of the Al-rich layer. The intensity profile of the GaSb quantum well (QW) region away from the nanoholes matches the designed 6.3 nm thickness. A slower intensity increase for *z* > 0 at QD locations arises from the Al-rich nanoring surrounding the nanoholes. Together, the AFM and STEM measurements provide consistent and complementary evidence for how Al coverage and growth temperature govern nanohole and QD morphology. These structural differences determine the exciton confinement, which we next correlate with the QD emission properties.

### Role of Nanohole Morphology in QD Emission

Wide-field fluorescence under nonresonant 780 nm LED excitation was imaged with an InGaAs camera. A long-pass filter was employed to suppress the LED scatter and QW background emission, isolating the QD signal (see Section S3 for setup details). Using a two-dimensional peak-finding algorithm with subpixel drift correction, we tracked 1850 QDs in *Sample A* and 510 QDs in *Sample B* within the camera’s field-of-view across varying temperatures (see Section S4 for methodology). The processed images confirmed spatially isolated, diffraction-limited spots across the field of view, consistent with the correlated random ordering of QDs expected from droplet-etched nucleation.^53,54^

Figure 2a summarizes the ensemble PL behavior extracted from these images. The QW emission quenches monotonically with increasing temperature, consistent with thermally activated escape into nonradiative channels and well described by an Arrhenius model. QDs in *Sample A*, associated with shallow nanoholes, exhibit nonmonotonic thermal behavior, with a pronounced maximum near 35 K and an additional low-temperature dip around 10 K (Figure 2a). Prior ensemble studies of GaSb QDs reported similar overall nonmonotonicity up to 10 K and explained it using a three-level rate-equation model incorporating the QW, a barrier, and the QD ground state.^47^ In this framework, the rise in QD intensity up to 35 K could be attributed to thermally activated injection from the QW across a barrier of ∼4 meV, while the quenching at higher temperatures is associated with excitonic escape back into the QW. These features point to stronger QD-QW coupling in *Sample A*. By contrast, QDs in *Sample B*, associated with deeper nanoholes, follow a trend more similar to the QW, showing only a weak dip around 7.5 K. This low-temperature feature in both samples suggests the presence of an additional scattering channel, necessitating an investigation of single QD emission.

**Figure 2:**
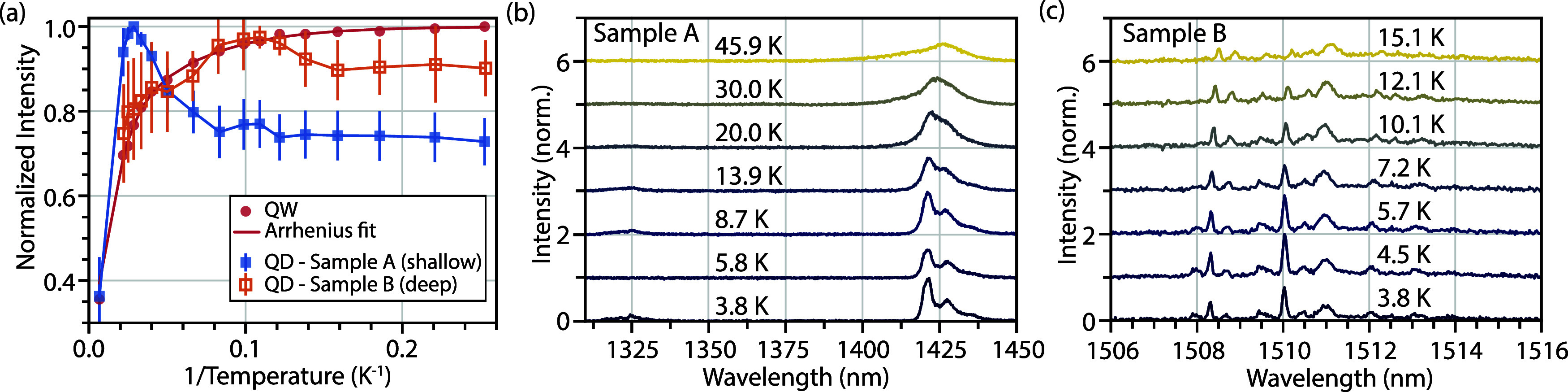
Temperature-dependent photoluminescence. (a) Normalized integrated intensity versus inverse temperature for the QW and QDs from both samples. Error bars indicate the spread across the measured ensemble (1850 and 510 QDs in Samples *A* and *B*, respectively). (b, c) μ-photoluminescence spectra of a single QD in each sample collected at different temperatures.

Representative single QD μ-PL spectra measured as a function of temperature (Figure 2b,c) further clarify these trends. In *Sample A*, most QDs exhibit broad, featureless spectra (≈20 nm line width), consistent with weak exciton localization and strong interaction with the environment. Only a small subset (<20%) shows partially resolved features narrower than 5 nm, similar to the representative spectrum in Figure 2b (see Section S5 for additional spectra and statistics). In contrast, QDs from *Sample B* display narrow, resolution-limited excitonic lines at low temperature when measured using a high-resolution spectrometer (45 μeV, 85 pm), confirming enhanced carrier confinement within deeper nanoholes. As the temperature increases, line width broadening becomes evident in both samples together with a modest change in intensity near 7–10 K, which can be attributed to intradot or phonon scattering processes. By ∼15 to 20 K, the emission spectra in both samples become featureless, but from very different starting points as discussed above.

These ensemble and single-QD measurements (36 QDs in *Sample A* and 77 in *Sample B*, see Section S5) together reveal how nanohole depth governs exciton localization and coupling to the surrounding quantum well. Shallow nanoholes enable rapid carrier exchange with the GaSb QW, producing broad, thermally activated emission, whereas deeper nanoholes suppress this coupling and yield stable, spectrally pure exciton lines. This morphological dependence is consistent with prior ensemble studies^47^ and motivates the time- and frequency-resolved measurements presented below, which directly probe the underlying carrier-transfer dynamics.

### Time- and Frequency-Resolved Signatures of QD–QW Coupling

The carrier dynamics underlying the morphology-dependent coupling were directly probed using wavelength- and time-resolved μ-PL of single QDs. Measurements on ten QDs in *Sample A* revealed consistent behavior, as illustrated for a representative emitter below. Figure 3a shows excitation power (*P*_exc_)-dependent spectra under pulsed nonresonant excitation. At low power (−27 dBm), the spectrum features a broad peak centered at 1431.7 nm with a full-width at half-maximum of 8.6 nm, indicative of weak QD exciton localization. With increasing *P*_exc_, additional blue-shifted spectral bands emerge sequentially at 1422.5 nm (5.6 meV shift) and 1413.0 nm, corresponding to dipole-allowed transitions between QD excited states.

**Figure 3:**
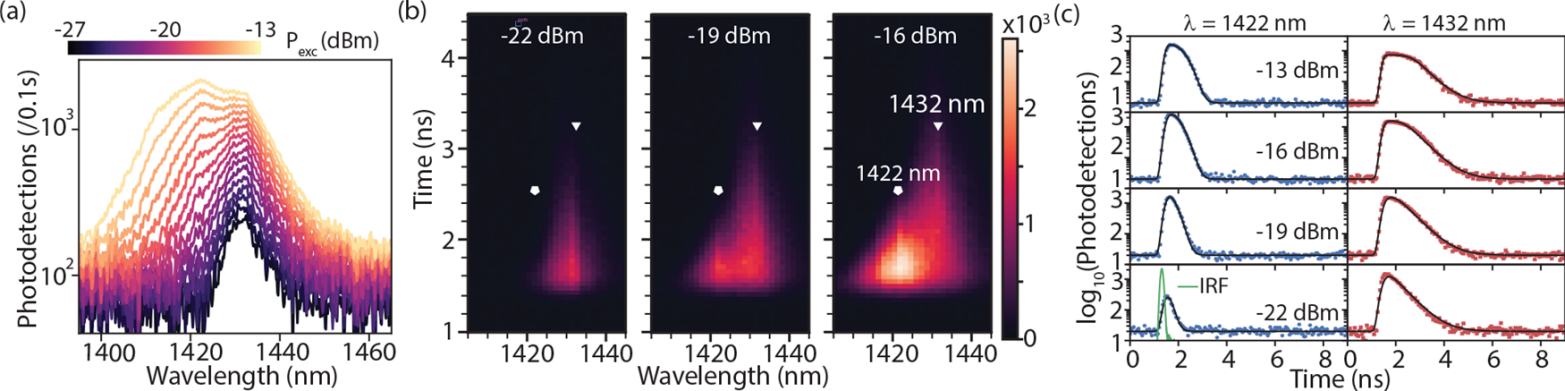
(a) Microphotoluminescence measured from a single QD in *Sample A* under above band gap excitation at varying pulsed laser average powers (*P*_exc_). (b) Time-wavelength maps of the emission at three representative excitation powers, highlighting the emergence of weak peak-like features around 1422 nm (diamond) and 1432 nm (triangle). (c) Time-resolved photoluminescence measurements at these two emission wavelengths [1422 nm (blue circles, left panel) and 1432 nm (red squares, right panel)] measured at different *P*_exc_. Black curves represent fits to a four-level model (see Section S7), including the detector instrument response function (IRF).

The gradual emergence of these features reflects Pauli-blocking-induced state filling, in which the saturation of lower-energy states leads to carrier accumulation in excited levels.^55,56^ Such behavior is rarely observed in single QDs due to slow carrier capture rates from the bulk or QW states. Previous demonstrations in tailored hybrid QD-QW nanostructures achieved enhanced transfer by adjusting QW thickness and barrier heights.^55,57,58^ Our AFM and STEM measurements (Figure 1) show that the deposited 6.3 nm GaSb overfills the nanoholes, creating a coupled QD-QW nanostructure. The absence of a tunneling barrier and the close energy alignment between the QW and QD states, as expected from electronic structure calculations,^47^ likely facilitate the rapid carrier transfer responsible for the observed state-filling features. Earlier work on GaSb/AlGaSb nanostructures reported ensemble-averaged spectral redistribution with increasing excitation power,^47^ and valley-dependent carrier capture dynamics in narrow-line width QDs,^45^ but neither directly resolved Pauli-blocking-driven state filling at the single-QD level. By contrast, our measurements track the sequential population of excited states within one QD and directly link them to the temporal dynamics below, a potential risk when overfilling GaSb QDs, which has not been explored in prior work.

Figure 3b displays time- and wavelength-resolved μ-PL maps at three representative excitation powers, highlighting the emergence of features at 1422 and 1432 nm. Time-resolved traces at these wavelengths (Figure 3c) exhibit nonmonotonic decays with plateaus at higher *P*_exc_, clear evidence of intersublevel relaxation bottlenecks. We employ a four-level rate equation framework to model these temporal dynamics (see Section S7 for details) incorporating QW–QD transfer (γ_tr_), QD intersublevel relaxation (γ_int_), and radiative decay rates (γ_QW_, γ_QD_). The model assumes incoherent carrier populations under nonresonant excitation and a uniform decay rate γ_QD_ for all QD states.^58^ We also neglect nonradiative losses as we could not identify a nonradiative decay within the uncertainty of an extended model that included γ_nrad_. This is consistent with the temperature-dependent, time-resolved PL measurements under weak excitation (*P*_exc_ = – 24 dB), which revealed the emergence of measurable nonradiative effects only at elevated temperatures (see Section S6). The bare QW decay rate, γ_QW_, was set based on independent measurements (see Section S6), while the pumping rate was calibrated to *P*_exc_. The instrument response function (IRF, Gaussian full-width at half-maximum of 130 ps; Figure 3c), including the excitation pulse width (2 ps), was explicitly included in the convolution during fitting. Global fitting of all eight datasets exhibits excellent agreement with the experiments, yielding the fit parameters γ_QD_ = 1.89 ± 0.05 ns^–1^, γ_tr_ = 11.2 ± 0.6 ns^–1^, and γ_int_ = 10 ± 2 ns^–1^. Although the fastest extracted rates approach the temporal resolution set by detector jitter, the global fitting approach, combined with the steep Gaussian roll-off of the IRF, allows reliable resolution of decay times down to ∼65 ps (rates of ∼15 ns^–1^).

The extracted QW-to-QD transfer time, 1/γ_tr_ ≈ 90 ps, is consistent with InGaAs QD-QW nanostructures,^57,58^ confirming that shallow-nanohole GaSb QDs act as hybrid QD–QW systems with ultrafast carrier exchange. This efficient injection, however, comes at the cost of poorly defined excitonic resonances, explaining the broad emission statistics of *Sample A* (Figure 2 and Section S5). Such broad emission spectra arising from parallel recombination and charge transfer channels inherently compromise photon antibunching as observed in GaN QDs operating at high temperature.^59,60^

### Efficient Telecom S-Band Quantum Emitters

Under above-bandgap CW excitation, single-QD μ-PL from *Sample B* exhibits spectrally narrow, resolution-limited lines near 1510 nm and no blue-shifted features at high power, indicating suppressed state filling and weak QW-QD coupling (Figure 4a). The emission intensity of the 1505.7 nm line (Figure 4a, inset) follows a saturation model *I*(*P*_exc_) = *I*_0_[1 – *e* ^–(*P*_exc_/*P*_sat_)^*b*^^], where *P*_sat_ is the saturation power in mW, and *b* is the stretching exponent quantifying the power dependence. The fitted exponent *b* = 1.18 ± 0.02 indicates a near-linear scaling consistent with a single-exciton recombination, with deviations reflecting additional relaxation or recombination channels.

**Figure 4:**
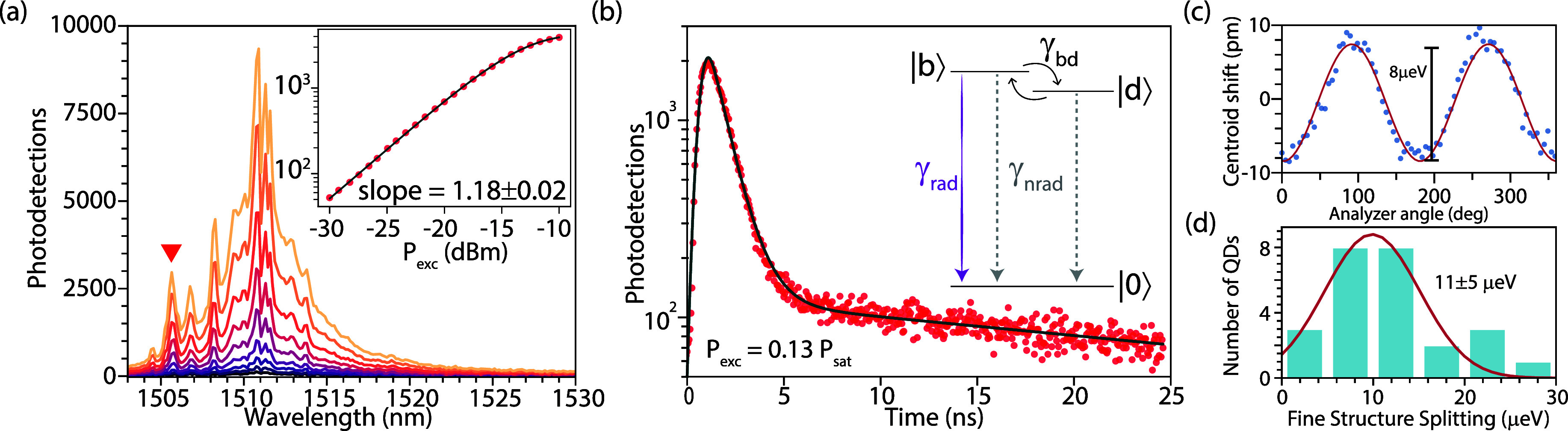
(a) Power-dependent PL spectra under above-bandgap excitation of a single QD in *Sample B*. The inset shows the power dependence of the peak (marked by a red triangle) fitted with a saturation model (red curve). (b) Time-resolved fluorescence of the same excitonic feature, fitted to a biexponential decay. The three-level exciton decay process is depicted in the inset. The fast and the slow decay components correspond to the decay of the bright and the dark excitons with decay rates γ_b_ = γ_rad_ + γ_nrad_ + γ_bd_ and γ_d_ = γ_nrad_ + γ_bd_, respectively. (c) Polarization-resolved spectroscopy of the same feature, showing centroid oscillations induced by fine-structure splitting (FSS). (d) Distribution of FSS measured across 25 QDs, with an average value of 11 ± 5 μeV.

Time-resolved μ-PL of the same line (see Figure 4b) reveals a biexponential decay, characteristic of bright |*b*⟩ and dark |*d*⟩ states of a neutral exciton (see inset for the three-level model). These states decay radiatively (γ_rad_) or nonradiatively (γ_nrad_) to the ground state |*g*⟩, with spin-flip processes occurring at a rate of γ_bd_ resulting in the biexponential time-dependent intensity:^61^

$$I(t)=A_{b}e^{-\gamma_{b}t}+A_{d}e^{-\gamma_{d}t}$$ 1

with the bright and dark exciton decay rates given by γ_b_ = γ_rad_ + γ_nrad_ + γ_bd_ and γ_d_ = γ_nrad_ + γ_bd_, respectively (see Section S8 for details). Assuming equal initial populations of bright and dark states under above-bandgap excitation, the extracted values of γ_b_ = 1.04 ± 0.02 ns^–1^ and γ_d_ = 0.020 ± 0.002 ns^–1^ indicate a 50-fold disparity. This significant decay rate disparity highlights a predominantly radiative recombination, quantified by the bright-state radiative branching ratio η ≡ (γ_b_ – γ_d_)/γ_b_ = 98 ± 1%.

Independent confirmation of the neutral exciton assignment is provided by polarization-resolved spectroscopy. Emission spectra collected at different rotation angles of a linear polarizer in the collection path were analyzed by fitting the 1505.7 nm line with a Gaussian. The fitted centroid exhibits oscillations arising from fine-structure splitting (FSS) of the neutral exciton (Figure 4c), which was modeled with a cosine to extract a splitting of 8 μeV. Taken together, the power-dependent scaling, biexponential lifetime, and FSS measurements unambiguously establish the observed emission line as a neutral exciton. Across 37 QDs, clear FSS-incuded oscillations were resolved in 25 with an average FSS of 11 ± 5 μeV (Figure 4d). In the remaining QDs, either the emission line was under-resolved or the splitting was below our resolution, consistent with ultrasmall FSS. Such small values are characteristic of droplet-etched QDs, which have also been observed in recent experiments on GaSb QDs,^45,46^ highlighting the potential for entangled-photon pair generation.

The exciton recombination dynamics were further investigated as a function of excitation power (Figure 5). The extracted bright-to-dark state amplitude ratio *A*_b_/*A*_d_ increases with *P*_exc_, indicating a higher fraction of photons emitted via radiative recombination. Concurrently, the dark-state decay rate γ_d_ also increases before saturating at 0.03 ns^–1^, consistent with enhanced spin-flip and nonradiative scattering processes at higher excitation power due to excess carrier–carrier scattering. Overall, the low γ_d_ and high branching ratio η = 98 ± 1% highlight GaSb QDs as promising telecom S-band emitters with minimal carrier scattering in the neutral-exciton manifold.

**Figure 5:**
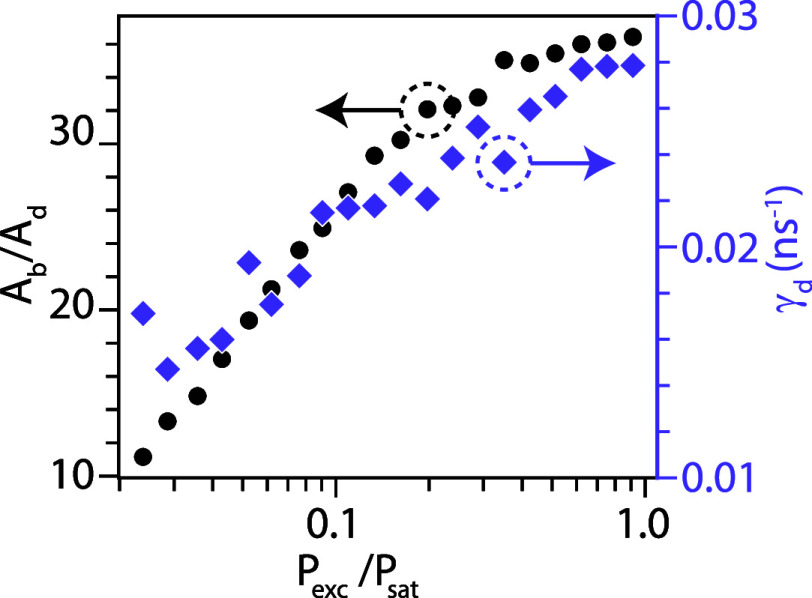
Power-dependent exciton recombination dynamics. The bright-to-dark state amplitude ratio (*A*_b_/*A*_d_) and the dark exciton decay rate (γ_d_) increases with laser power.

### Single-Photon Emission under Nonresonant and Resonant Excitation

To evaluate single-photon emission, we performed Hanbury-Brown Twiss (HBT) interferometry on the neutral exciton analyzed in Figures 4 and 5. The time-resolved coincidence histogram at *P*_exc_ = 0.13*P*_sat_ (Figure 6) shows a pronounced antibunching dip at τ = 0, confirming single-photon emission. A weak bunching feature for |τ| < 2 ns indicates multiphoton contributions induced by nonresonant excitation,^62^ arising from carrier capture from the barrier layer into the QD with a characteristic rate γ_cap_. When γ_cap_ is comparable to or faster than the radiative rate, multiple carriers may be trapped in the QD, leading to multiphoton emission.

**Figure 6:**
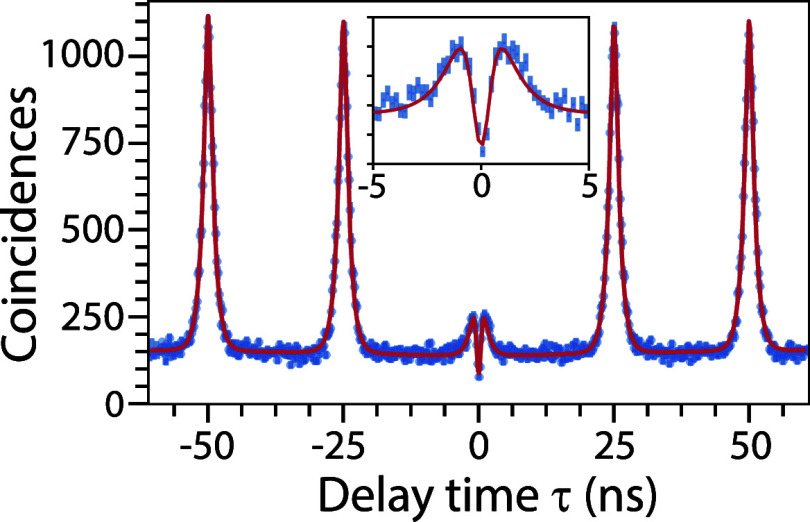
Hanbury-Brown and Twiss interference (HBT) measurement of the exciton emission under pulsed above-bandgap excitation at *P*_exc_ = 0.13*P*_sat_, showing antibunching with *g* ^(2)^ = 0.18 ± 0.05. The inset shows a zoom around τ = 0.

We modeled the coincidence histogram using a rate-equation approach that incorporates bright and dark states as well as carrier capture:^63–65^

$$N_{c}(\tau )=C_{0}\left[e^{-|\tau |\gamma_{b}}+\frac{A_{d}}{A_{b}}e^{-|\tau |\gamma_{d}}\right]-B_{0}e^{-|\tau |\gamma_{\mathrm{cap}}}+C_{1}\sum_{n\neq 0}\left[e^{-|\tau -nt_{\mathrm{rep}}|\gamma_{b}}+\frac{A_{d}}{A_{b}}e^{-|\tau -nt_{\mathrm{rep}}|\gamma_{d}}\right]+C_{b}$$ 2

where *B*_0_ accounts for capture-induced coincidences, *C*_b_ denotes the background, and *C*_0_ and *C*_1_ correspond to coincidence events at τ = 0 and τ = *nt*_rep_ (*n* ≠ 0), respectively. Fitting with γ_b_ and γ_d_ fixed to values from time-resolved PL (Figure 4) yields γ_cap_ = 3.26 ± 0.01 ns^–1^. The single-photon purity, defined as the ratio of the fitted peak area at τ = 0 to the average peak area at τ = *nt*_rep_, gives *g* ^(2)^ = 0.18 ± 0.05.

We note that previous work on GaSb QDs attributed similar *g* ^(2)^(τ = 0) features to Γ-*L* intervalley scattering.^45^ However, those studies also reported charge transfer rates nearly an order of magnitude slower than the exciton radiative rate, inconsistent with the fast capture (γ_cap_ ≳ γ_b_) required to produce such a feature at τ = 0. Furthermore, while their time-resolved PL displayed pronounced state filling, our power-dependent time-resolved measurements (Figure 4b and Section S9) reveal a clean biexponential decay up to *P*_sat_. Together, these results establish a self-consistent interpretation of the weak multiphoton contribution as arising from extrinsic carrier capture. Importantly, the near-vanishing coincidences at τ = 0 confirm the high intrinsic purity of GaSb QD emission, underscoring the need for mitigating secondary carrier capture.

Resonant excitation of QD states mitigates secondary carrier capture and thereby improves single-photon purity. We performed phonon-assisted quasi-resonant excitation using sub-5 ps pulses from a filtered supercontinuum source (see Section S3b for methodology). The role of excitation conditions is evident in the μ-PL spectra (Figure 7a), contrasting nonresonant above-bandgap excitation and quasi-resonant excitation with a laser tuned to 1476 nm (17 meV above the neutral exciton line marked with a green triangle). While above-band excitation produces a broad background from carriers generated in the barrier layer, quasi-resonant excitation selectively addresses the QD states and yields isolated, resolution-limited (45 μeV) peaks. Note that the above bandgap spectrum was recorded with a lower resolution spectrometer (110 μeV).

**Figure 7:**
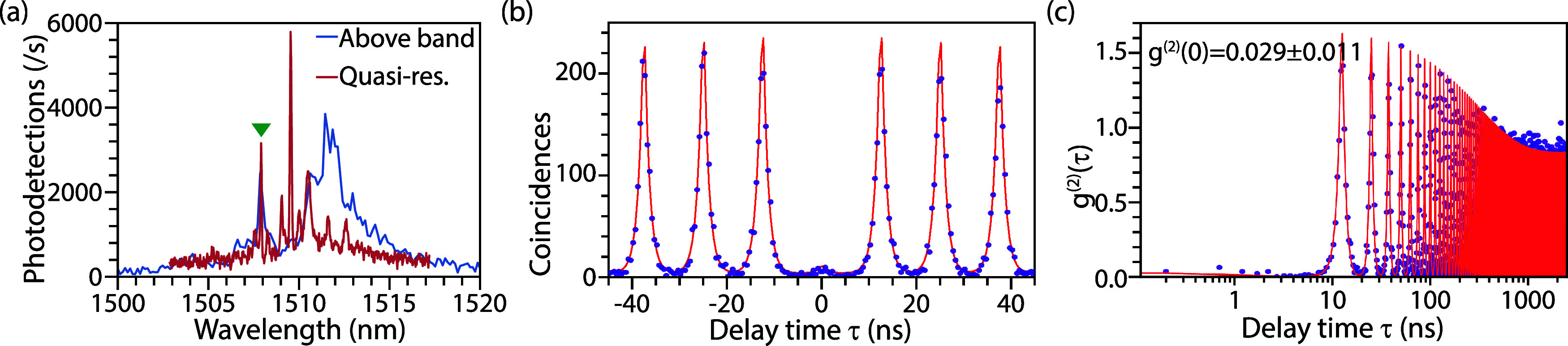
Quasi-resonant excitation of GaSb QDs. (a) μ-PL spectra of a QD under above-band (blue) and quasi-resonant excitation (red), highlighting suppression of background states. HBT measurement of the emission peak (blue triangle) in linear (b) and logarithmic (c) time scales together with fits, highlighting near-ideal antibunching at short delays and blinking on longer (310 ± 40 ns) time scales.

The benefit of quasi-resonant excitation is confirmed by HBT measurements (Figure 7b,c) at *P*_exc_ = 0.15*P*_sat_, which exhibit a pronounced antibunching dip at τ = 0. A raw estimate of *g* ^(2)^(τ = 0) ≈ 0.015 obtained from the ratio of coincidences under the peaks at τ = 0 and τ = 12.5 ns. Extending the measurement window to |τ| = 2500 ns reveals a slow blinking component that was not observed under above-band excitation. Fitting the coincidence histogram with a multi-timescale blinking model,^66^ we reproduce the data across the full range and extract a blinking time scale of 310 ± 40 ns (Figure 7c).

From the fitted model, we extract *g* ^(2)^(0) = 0.029 ± 0.011, representing the lowest value reported to date for GaSb-based QDs in the telecom band. In contrast to the weak multiphoton contribution observed under above-band excitation (Figure 6), quasi-resonant pumping suppresses carrier capture and state-filling effects, thereby accessing the high intrinsic single-photon purity of GaSb QDs.^46^ The microscopic origin of the blinking remains unclear, though the time scales suggest charge or spin fluctuations in the QD environment. Importantly, this long-time-scale blinking does not significantly degrade the short-time antibunching, and the single-photon purity is comparable to the state-of-the-art demonstrations in InGaAs QDs.

## Conclusions

In summary, we have demonstrated single-photon emission from GaSb/AlGaSb QDs grown via nanohole etching, establishing their potential as efficient telecom-wavelength quantum light sources. Structural and optical characterization of single QDs revealed a direct correlation between nanohole morphology, charge-transfer dynamics, and exciton recombination. QW-QD coupled structures formed by infilling shallow nanoholes possess ultrafast carrier transfer (10 ns^–1^) that strongly limits emission coherence. In contrast, QDs formed by infilling deeper nanoholes exhibited clean exciton features with near-unity (98 ± 1%) bright-to-dark state branching ratio, competitive with state-of-the-art InGaAs QDs while extending operation into the telecom band.^10,11^ HBT measurements confirmed robust single-photon emission, with photon purity improving from *g* ^(2)^(0) = 0.18 ± 0.05 under nonresonant excitation to 0.029 ± 0.011 under pulsed quasi-resonant excitation. The suppression of secondary carrier capture highlights the central role of the excitation mechanism and establishes GaSb QDs as high-purity quantum emitters in the telecom S-band. Additionally, polarization-resolved spectroscopy revealed ultrasmall fine structure splitting (11 ± 5 μeV) suitable for entangled-photon pair generation employing the biexciton cascade.

Our results provide a foundation for developing scalable, telecom-band spin-photon interfaces for quantum networks. The recent success of local droplet etched GaAs QDs in achieving record-long spin coherence and efficient spin cooling^42,43^ suggests that GaSb QDs could realize coherent spin-photon interfaces at telecom wavelengths. Future efforts should focus on charge-state control via diode heterostructures,^67,68^ direct measurements of spin coherence, and photonic integration to realize key building blocks for fiber-based quantum networking, such as indistinguishable photon sources and spin-photon entanglement.

## Experimental Methods

### STEM Analysis

A thin lamella for STEM analysis was prepared via Xe^+^ plasma-focused ion beam milling in a Helios 5 UXe (Thermo Fisher Scientific). An initial layer of electron beam-induced deposited carbon protects the surface from ion damage, followed by a thicker layer of ion beam-induced tungsten deposition. A region was lifted out and welded to a copper grid, then thinned with progressively lower ion beam voltages and currents until the sample was electron transparent.

STEM analysis was performed at 300 kV accelerating voltage in a Spectra Ultra (Thermo Fisher Scientific) equipped with an X-FEG/UltiMono source and a probe- and image-corrector. Images were acquired utilizing 28 mrad convergence semiangle and approximately 100 pA beam current, with HAADF inner and outer angles of 49 and 200 mrad, respectively.

### Optical Measurements

Details of the experimental setup are provided in Supporting Information Section S2.

## Supplementary Material



## Notes

HAADF-STEM images of *Sample A*, statistical characterization of nanoholes, optical setup for microphotoluminescence, wide-area, temperature-dependent fluorescence, single QD emission statistics, spatially resolved emission spectra, four-level rate equation model of coupled QD-QW nanostructures, three-level rate equation model of bright-dark QD exciton dynamics, power-dependent time-resolved μ-PL on *Sample B* (PDF)
