# Supplementary material for: Engineering Nanohole-Etched Quantum Dots for Telecom-Band Single-Photon Generation
Source: ACS Nano. 2026 Jan 9;20(3):2872–80. doi: 10.1021/acsnano.5c17982 (PMC12854758; doi:10.1021/acsnano.5c17982)
Supplement: Supplementary file 1 [file nn5c17982_si_001.pdf]

Supplementary Information: Engineering Nanohole-Etched Quantum Dots for Telecom-Band Single-Photon Generation

Ian M. Masson,† Aden Hageman,† Caleb Whittier,‡ David Montealegre,† Bhaveshkumar Kamaliya,‡ Nabil D. Bassim,‡,¶ John P. Prineas,† and Ravitej Uppu*,†

†*Department of Physics & Astronomy, The University of Iowa, Iowa City, IA 52242*

‡*Department of Materials Science & Engineering, McMaster University, Hamilton, Ontario L8S 4L7, Canada*

¶*Canadian Centre for Electron Microscopy, McMaster University, Hamilton, Ontario L8S 4M1, Canada*

E-mail: ravitej-uppu@uiowa.edu

Phone: +1 (319) 467-3201

S1. HAADF-STEM Images of Sample A

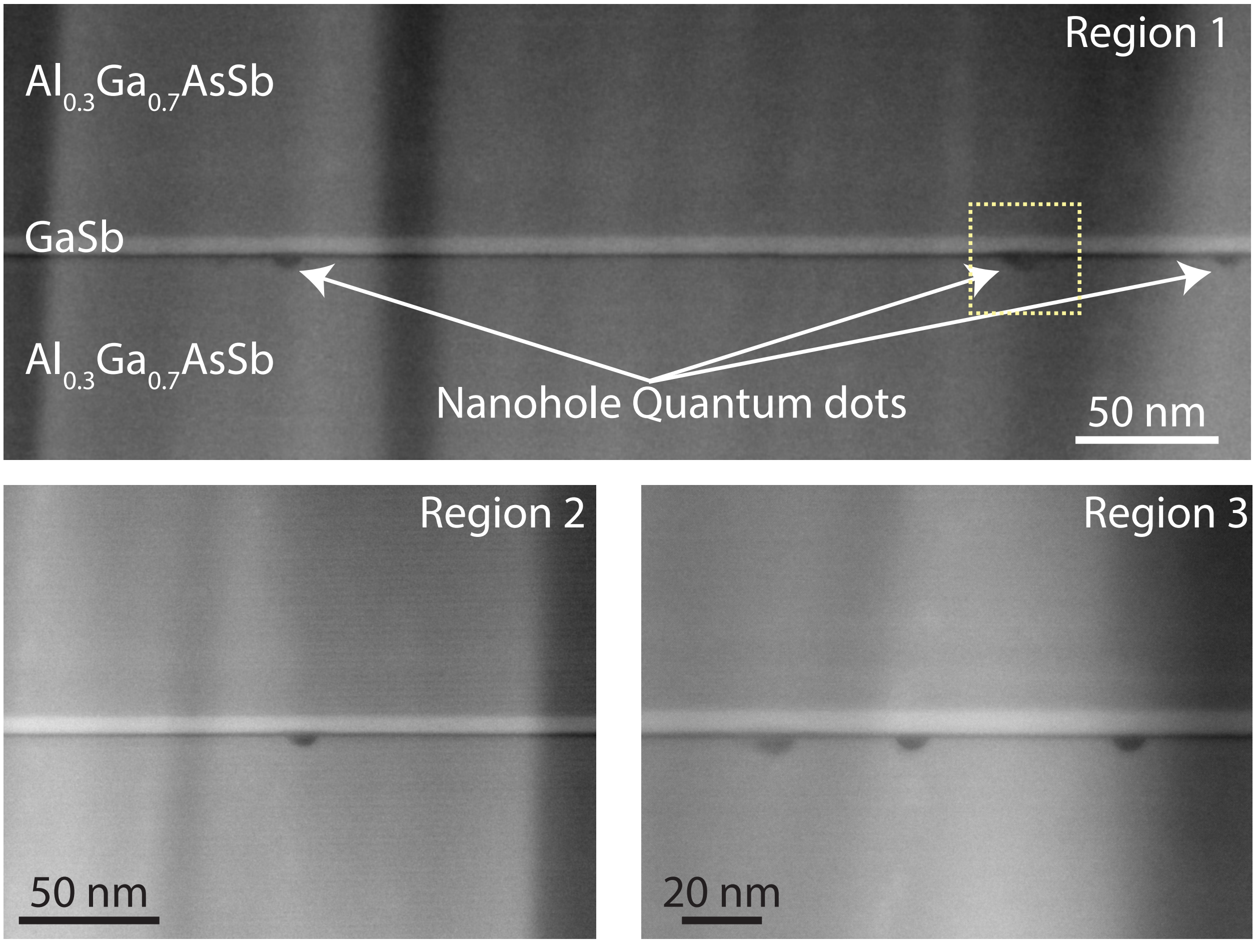

Figure S1: HAADF-STEM images from three distinct sample regions, prepared using Xe+ plasma focused ion beam milling. These wider span images reveal uniform epitaxial layers, which is evident from the thin GaSb quantum well layer. Quantum dots (QDs) were observed in all three regions of *Sample A*, consistent with its high nanohole density. The yellow dotted box in Region 1 highlights the overlapping QD region discussed in the main text. In contrast, other QDs appear shallow ($\leq$5 nm) and do not overlap in the sample cross-section, indicating that the sample predominantly contains a high density of shallow nanoholes along with a lower density of deeper nanoholes.

S2. Statistical Characterization of Nanoholes

The morphology of droplet-etched nanoholes in both samples was examined using atomic force microscopy (AFM). Nanohole characterization samples were grown by MBE on one-quarter sections of a 2" epi-ready GaSb wafer, as described in the main text. Each quarter wafer was cleaved into three pieces, and AFM scans were performed on 50×50 µm^2 regions near the center of each piece to minimize artifacts from scribe dust. AFM measurements were carried out using a Nanosurf NaioAFM equipped with a super-sharp tip (Nanosensors SSS-NCLR, radius <5 nm, high aspect ratio), ensuring the accurate capture of nanohole profiles. Representative data are shown in Figure S2, which includes wide-area scans (right panels) and zoomed-in views of individual nanoholes (left panels). Table S1 summarizes the areal densities and size distributions extracted from three regions analyzed per sample (approximately 30 nanoholes in total, i.e., 10 per cleaved wafer piece).

Table S1: Areal density and size statistics of nanoholes in Samples A and B.

Sample	Nanohole type	Areal density (µm^{-2})	Average Relief (nm)
A	Deep nanoholes	0.19	11 ± 2
	Shallow nanoholes	> 10	2.5 ± 1.0
B	Deep nanoholes	0.04	22 ± 3

For Sample A (3.2 ML Al, 395 °C; Figure S2(a)), AFM scans reveal both deep nanoholes with reliefs exceeding 8 nm and a large number of much shallower depressions (<4 nm), resulting in a clear bimodal depth distribution. In contrast, Sample B (2.5 ML Al, 410 °C; Figure S2(b)) shows only well-defined deep nanoholes with average reliefs of 22 ± 3 nm at significantly lower areal density, without a shallow population. These AFM-derived statistics are corroborated by STEM analysis presented in the main text (Figure 1). In *Sample A*, HAADF-STEM images resolved infilled nanoholes of differing depths (e.g., QD1 and QD2 at 5 and 8 nm in Figure 1 of main text), consistent with the bimodality observed by AFM. Both AFM and STEM indicate the presence of nanorings, approximately 3 nm in height, surrounding the nanoholes, resulting in a total nanohole relief of around 11 nm. Analysis of

the nanohole profiles shows (112)-type sidewall faceting with an angle of $35 \pm 2°$, in excellent agreement between AFM line profiles and cross-sectional STEM.

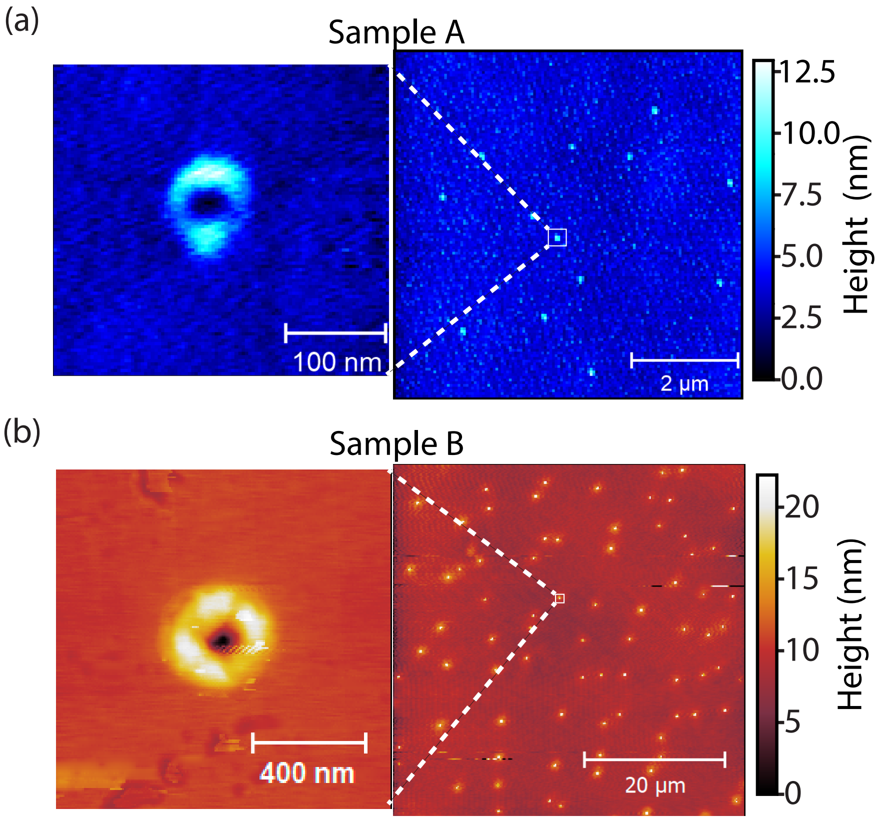

Figure S2: Representative AFM images of nanoholes formed by Al droplet etching. **(a)** Sample A (3.2 ML Al, 395 °C): the high-resolution image (left) shows a nanohole with a relief of 12 nm, while the larger-area scan (right) reveals a bimodal distribution featuring a lower density of nanoholes with reliefs over 8 nm together with a high density of shallow depressions (below 4 nm), which appear as bright pixels in the wide-area image. **(b)** Sample B (2.75 ML Al, 410 °C): the high-resolution image (left) illustrates a single deep nanohole, and the overview scan (right) shows a uniform distribution of deep nanoholes with average reliefs of 22 ± 3 nm at significantly lower areal density, without evidence of a shallow nanohole population.

The AFM and STEM measurements together demonstrate a clear correlation between growth conditions and nanohole morphology. High Al coverage at moderate temperature (Sample A) produces a bimodal depth distribution with enhanced areal density. In contrast, lower Al coverage at slightly elevated temperature (Sample B) yields fewer, uniformly deep nanoholes. These structural differences set the initial conditions for the density, uniformity, and confinement environment of the subsequently formed GaSb quantum dots.

S3. Optical Setup for Micro-Photoluminescence

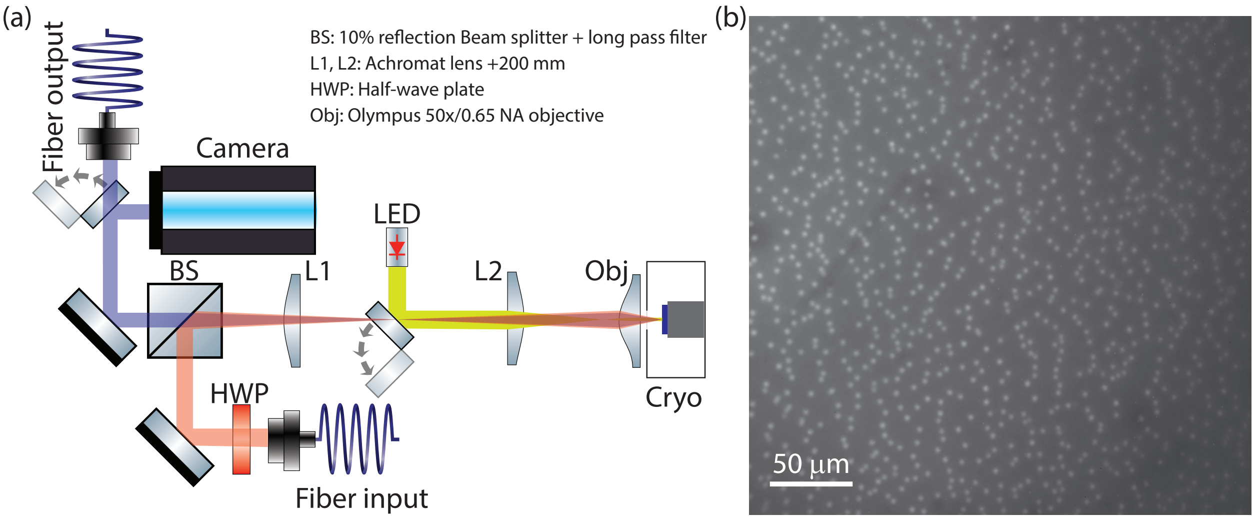

Figure S3: (a) Schematic of the experimental setup for optically characterizing quantum dots. Excitation light from a laser is collimated at the "Fiber input". The emission collected by Obj is routed to the single-mode fiber at the "Fiber output". The LED and the InGaAs Camera are employed to locate QDs in the field of view of the sample cooled to 3.5 K in a cryostat. A 10/90 beamsplitter BS and a long pass spectral filter separate the excitation and collection. (b) shows a wide-field fluorescence image captured with a long pass filter on the camera when the sample was illuminated using the 780 nm LED. The bright diffraction-limited spots are single QDs, each of which can be addressed using a focused laser beam.

Micro-photoluminescence experiments were conducted on samples cooled to 3.5 K in a closed-cycle helium cryostat (Montana Instruments Cryocore). The optical setup is schematically illustrated in Fig. S3a. The sample was accessed through a high-numerical-aperture microscope objective (Obj; Olympus LCPlan N 50x/0.65 IR) via a 0.5 mm-thick optical window on the cryostat (Edmund Optics sapphire window with NIR II anti-reflection coating). The fiber-coupled excitation laser was collimated using a 20 mm-focal-length achromatic lens (Schäfter+Kirchhoff 60FC-4-M20-37), producing an excitation beam with a 4.2 mm diameter to nearly fill the objective's back aperture. The objective focused the laser beam to a diffraction-limited 1 µm spot on the sample. To enable laser spot translation on the sample, a pair of achromatic lenses (L_1 and L_2, each with a +200 mm focal length) imaged the laser turning mirror onto the back focal plane of Obj. The fluorescence from the sample

is collected using Obj and routed back collinearly with the excitation path. A beam splitter (BS; Thorland BSN12R; 10% reflection: 90% transmission) separated the collected emission from the excitation laser. The transmitted emission passed through a long-pass filter before being coupled into a single-mode optical fiber via a fiber collimator (focal length 20 mm), acting as a spatial filter to collect light selectively from the diffraction-limited focus. Fluorescence from the sample can also be imaged onto an InGaAs camera. Figure S3b shows a wide-field fluorescence image of the sample when excited using a 780 nm LED. The bright diffraction-limited spots in the image, captured with a 1450 nm long pass filter to reject the laser and suppress the QW emission, correspond to single QDs.

3.1 QD Emission Collection & Characterization

The fiber output was directed to either a spectrometer (Horiba iHR550; 300 lines/mm and 950 lines/mm gratings) with an InGaAs camera (Andor iDus 490A) for rapid spectral acquisition or a home-built monochromator (900 lines/mm grating) for photon-counting experiments. The monochromator-filtered emission was relayed via a single-mode fiber to superconducting nanowire single-photon detectors (SNSPDs; PhotonSpot), operating at 800 mK (PhotonSpot), with a timing jitter of less than 100 ps.

For time-resolved photoluminescence measurements, photon detection events and the laser clock signal were recorded using time-correlation electronics (Swabian TimeTagger Ultra) with channel-to-channel timing jitter below 10 ps. Time-resolved second-order coherence measurements were conducted similarly by splitting the spectrally filtered emission via a 50/50 non-polarizing fiber coupler. The two outputs were detected by separate SNSPDs, and time-correlation electronics processed the detection events to generate time-delay histograms of coincident photon arrivals.

The instrument response function (IRF) was measured by directing an attenuated ~ 2 ps laser pulse to each of the detectors, and measuring the time-resolved histogram with a 1 ps detection time step. The measured histogram is shown in Figure 3c and Figure S6b, which

was fit to a Gaussian function, yielding a full-width at half-maximum (FWHM) of ≈ 130 ps for each detector. The IRF for the second-order coherence measurements was constructed similarly by splitting the laser pulse using a non-polarizing fiber beam splitter and measuring the time-resolved coincidence histogram. All fits to the time-resolved PL and second-order coherence measurements were numerically convolved with these measured IRFs.

3.2 Excitation lasers

Above band excitation was provided by a ytterbium fiber oscillator emitting 1030 nm pulses of 4 ps duration at a 40 MHz repetition rate. Continuous-wave quasi-resonant excitation of the QDs was performed using a tunable diode laser (EXFO Osics T100-1520) to identify the resonant excitation wavelengths. The polarized laser output was made diagonal by adjusting the half-wave plate in the excitation path.

Pulsed quasi-resonant excitation was implemented using a home-built tunable supercontinuum source as described below. Emission from a Ti:Sapphire laser oscillator (Spectra Physics Tsunami; tuned to 810 nm; 90 fs pulse length; 80 MHz repetition rate) was focused into a nonlinear photonic crystal fiber (NKT FemtoWHITE 800) using a microscope objective to match the fiber mode-field diameter (average power around 350 mW). The generated supercontinuum light is collimated using an off-axis parabolic mirror (Thorlabs MPD00M9-P01) and incident on a 950 nm short-pass dichroic beam splitter. The reflected light from the dichroic is further cleaned using a 950 nm long-pass filter and incident on a 900 lines/mm diffraction grating mounted on a rotation stage (Thorlabs ELL18). The diffracted first-order light is collected using an achromatic fiber collimator (Schäfter+Kirchhoff 60FC-4-M20-37) into a polarization-maintaining fiber patchcord.

3.3 Setup collection/detection efficiency

The net probability that an emitted photon produces a detection event is a product of collection (η_{coll}) and transmsision efficiencies of the optical setup and the detection efficiency

of the detector (η_{det}) given by the relation: $\eta_{\text{coll}} \times T_{\text{obj}} \times T_{\text{optics}} \times T_{\text{filter}} \times \eta_{\text{det}}$. The geometric collection efficiency of a dipole emitter embedded in a high-index medium (here $n \approx 3.4$ at 1.5 µm wavelength) and collected using an air-immersion high-NA objective is limited by the extraction efficiency $\sim 1/(4n^2)$. The objective collects a fraction of this light depending on the critical angle $\theta_c = \arcsin(1/n)$ and the collection angle $\theta_o = \arcsin[(1/n)\text{NA}]$. We assume a planar, unpatterned surface, which serves as a conservative lower bound without any nanophotonic enhancement. For the 0.65 NA objective, the estimated η_{coll} is around 0.9%. The measured transmittance of the objective across the spectral range of interest using a tunable laser was found to be $T_{\text{obj}} = 62 \pm 5\%$. The cumulative transmittance of optics, which includes the AR-coated sapphire window, telescope lens, beam splitter, long pass filter, and the fiber collimator, was similarly measured using a tunable laser to be $T_{\text{optics}} = 72 \pm 3\%$. We employ a grating monochromator with fiber in-/out-coupling whose end-to-end efficiency is $T_{\text{filter}} = 35\%$. The measured detection efficiency of the SNSPD was $\eta_{\text{det}} = 80 \pm 7\%$. Thus, the total end-to-end efficiency of the setup is $\approx 0.1\%$, primarily limited by the geometric collection efficiency.

S4. Wide-area, Temperature-Dependent Fluorescence

To distinguish between fluorescence originating from individual QDs and background emission from the surrounding QW, we performed wide-field fluorescence imaging under continuous-wave 780 nm LED excitation using different long-pass filters. Representative images for both samples are shown in Figure S4.

4.1 Sample Comparison

For *Sample B* (deep nanoholes, $\rho \approx 0.04$ µm^{-2}), wide-field imaging with a 1000 nm long-pass filter (Figure S4a) reveals a strong QW background with superimposed QD spots. Switching to a 1450 nm filter (Figure S4b) efficiently suppresses the QW contribution, leaving

isolated, diffraction-limited QDs. Their spatial separation confirms suitability for single-emitter spectroscopy.

In contrast, *Sample A* (shallow, overfilled nanoholes, $\rho \approx 0.15\ \mu m^{-2}$) exhibits a much higher density of bright emitters (Figure S4c). Although the sample also features a higher density of shallow nanoholes, they either do not emit or overlap spectrally with the QW. Thus, the majority of bright centers remain distinguishable within our resolution limit, as discussed below.

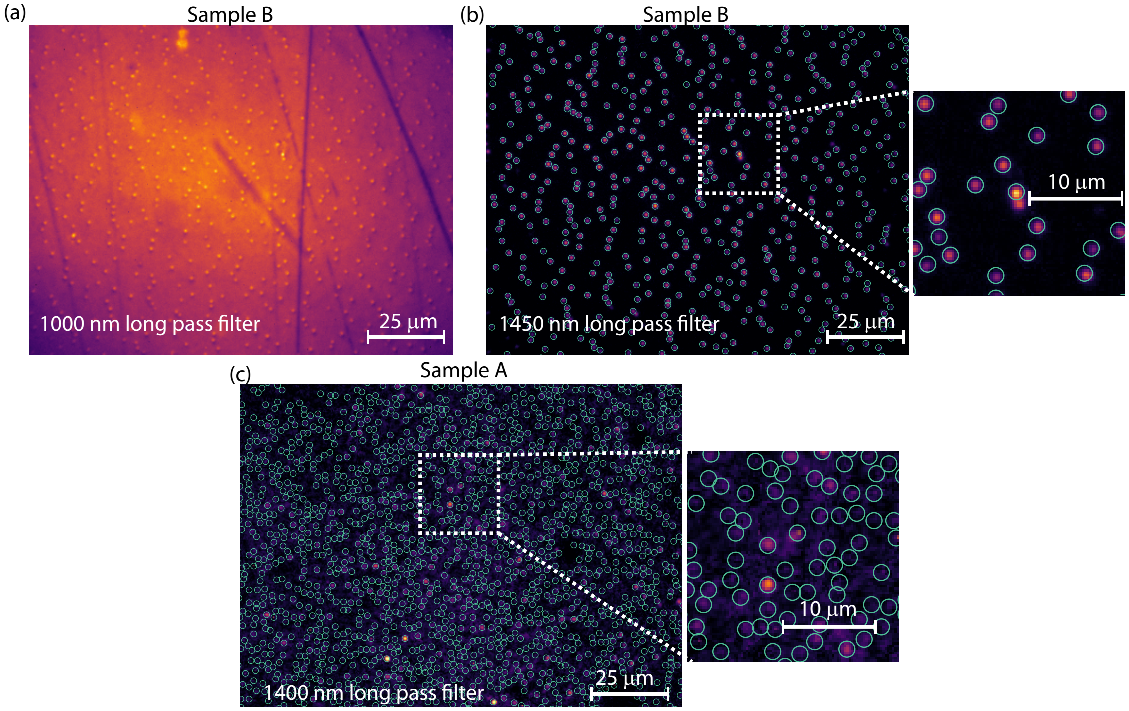

Figure S4: Wide-field fluorescence imaging of quantum dots (QDs) under 780 nm LED excitation. **(a)** *Sample B* imaged with a 1000 nm long-pass filter shows strong background emission from the quantum well (QW). **(b)** The same region imaged with a 1450 nm long-pass filter efficiently suppresses the QW contribution, revealing spatially isolated, diffraction-limited QD emission spots (green circles: 2D peak-finding analysis of QD locations). **(c)** Wide-field imaging of *Sample A* using a 1400 nm long-pass filter shows a much higher areal density of emitting centers. Insets in (**b**,**c**) highlight representative regions at higher magnification. These wide-field maps confirm that individual QDs can be clearly isolated for statistical analysis.

4.2 Optical Resolution & QD Density Analysis

At emission wavelengths around 1400−1500 nm, the diffraction-limited lateral resolution according to the Abbe criterion is $d = \lambda/(2\,\mathrm{NA}) \approx 1.1$ µm,with λ the emission wavelength and numerical aperture NA of 0.65. On the detector (Photonic Science PSEL VGA, 15 µm pixels) with an effective 55× optical magnification, each pixel corresponds to 0.27 µm in object space. The diffraction-limited spot therefore spans ~ 4 pixels, satisfying the Nyquist sampling criterion and enabling reliable localization of point sources.

For a randomly distributed array of QDs, the spatial location can be modeled as a Poisson process of areal density ρ. The probability that a given QD has no neighbors within the diffraction-limit d (i.e., appears isolated/resolved) is the void probability $P_{\mathrm{resolved}} = \exp(-\rho\pi d^2)$. At the areal nanohole densities measured for samples (cf. Table S1), we expect the probability of resolving individual dots to be 47% and 86% for *Sample A* and *B*, respectively.

Real droplet-etched nanoholes, however, deviate from purely random (Poisson) statistics. Surface diffusion and nucleation dynamics impose spatial correlations that suppress the formation of near-coincident neighbors.[1] This short-range repulsion is well described by Voronoi cell area statistics following a generalized Wigner distribution.[2,3] At densities of $\rho \approx 0.19$ µm^{-2}, typical Voronoi cell areas are ~ 5 µm^2.[4] Assuming a circular Voronoi cell, we can calculate the corresponding effective exclusion radius $r_h \sim 1.3$ µm, as the region where only one QD can be found. A simple hard-core corrected Poisson model estimates the probability of finding two QDs closer than d as $P_{\mathrm{resolved}} \approx \exp\big[-\rho\pi(d^2 - r_h^2)\big]$, when $d > r_h$. Given that the optical resolution d is comparable to or smaller than the estimated r_h, we anticipate clearly resolving single QDs in both samples (Figure S4).

4.3 Fluorescence Intensity Analysis

The fluorescence images are analyzed using a 2D peak-finding algorithm using scikit-image in Python. Intensity in 3×3 pixel (i.e., 0.8×0.8 µm^2) area around the identified peaks is used to analyze the intensity of single QDs. The identified QDs in each image are shown as green circles in Fig. S4. To ensure consistent tracking during temperature-dependent measurements, we employed an image-correlation algorithm to correct for minor stage drifts, thereby allowing for the reliable identification of the emitters across successive temperature steps. In total, the wide-field fluorescence imaging approach enabled tracking 1850 and 510 QDs in Samples *A* and *B*, respectively. Thus, this procedure enabled robust statistical comparison of QD emission evolution with temperature, directly linking the wide-field maps to the spectral trends shown in the main text (Figure 2). Note that we measured only ± 2 pixel drifts across the whole temperature range of our analysis due to the inherent low thermal drift of the sample platform.

By comparing *Samples A* and *B*, we correlate wide-field fluorescence maps with temperature-dependent spectra (Figure 2) and structural data (Table S1). Shallow nanohole QDs (*Sample A*) exhibit rapid thermal quenching and broad, inhomogeneous emission, while deep nanohole QDs (*Sample B*) retain bright, sharp, and discrete peaks. This consistent correspondence confirms that nanohole morphology critically governs the optical behavior of the resulting QDs.

Earlier work has primarily inferred the QW-QD coupling and the role of nanohole morphology from ensemble-averaged spectra.[5] Here, our imaging-based approach allows us to track hundreds to thousands of individual QDs, offering access to sample heterogeneity and enabling future studies that directly map morphological capture area (e.g., Voronoi cells) to emission properties.[4]

S5. Single QD Emission Statistics

To address dot-to-dot variability beyond the few representative cases in the main text, we quantified emission statistics from tens (>35) of QDs. Figure S5 shows spectra of around 10 QDs per sample. Table S2 summarizes the ensemble statistics quantitatively of emission center wavelength, linewidths, and inhomogeneity. In *Sample A* (shallow-nanohole infilled GaSb QDs), the average peak wavelength of single QD emission was found to be 1422 $\pm$ 12 nm. Most QDs exhibited broad, almost featureless emission spectra with a full-width at half-maximum (FWHM) around 25 nm, and only a small fraction (<10%) show any discernible, yet broad, exciton features. As discussed in the main text, this behavior is consistent with QD-QW hybridization and strong state-filling behavior reported in our time- and frequency-resolved measurements in the main text. We carried out time- and frequency-resolved measurements on 10 QDs and observed similar behavior as discussed in the main text.

Table S2: Statistics of emission spectra.

**Metric**	**Sample A**	**Sample B**
QDs analyzed	36	77
Emission center (nm)	1422	1509
Emission spectrum span (nm)	≈ 40	≈ 10
Inhomogeneity (meV)	$10^{\dagger}$	$5.3 \pm 0.5^{\ddagger}$
Linewidth (μeV)	–	≤ 50 (resolution-limited)
QDs with spectral features	$< 20\%$	$> 90\%$
Neutral exciton identified*	N/A	$> 50\%$

† Defined as the spectral distribution of the centroid of the emission spectrum.
‡ Defined as the FWHM of the spectral distribution of the first well-defined emission peak (or the location of the neutral exciton, if identified).
* Positive identification of the neutral exciton is defined as the observation of fine-structure-splitting-induced oscillation of an emission line.

In Sample B (deeper nanoholes), the average emission frequency is centered at 1509 nm with a much smaller inhomogeneous spread <5 meV. This low inhomogeneity is consis-

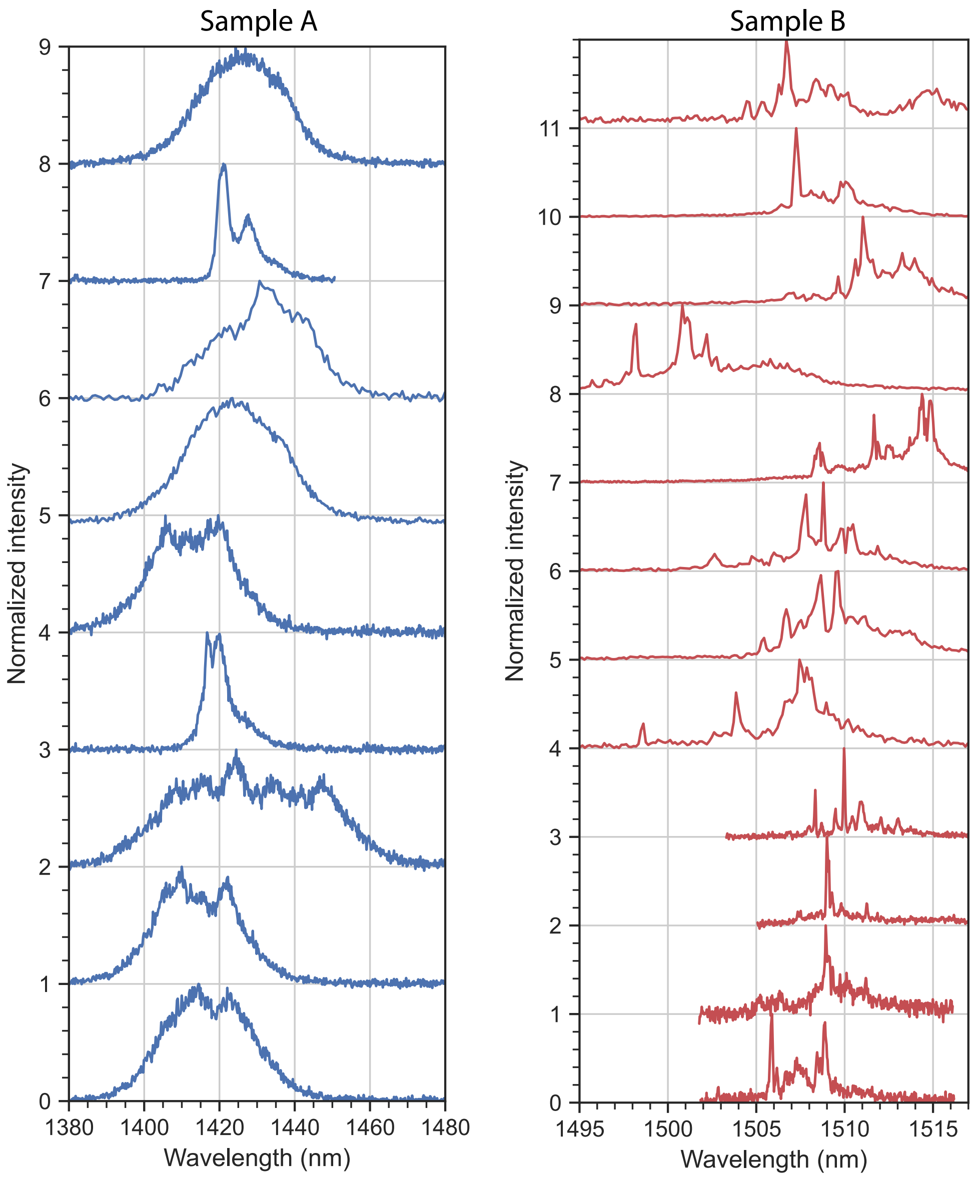

Figure S5: Random selection of single-QD spectra from *Sample A* (left; GaSb QDs formed by infilling shallow nanoholes) and *Sample B* (right; deeper nanoholes). *Sample A* typically shows broad emission without a clear excitonic structure. *Sample B* displays narrow, well-resolved excitonic lines (resolution-limited). Note that the bottom four spectra of *Sample B* were captured with the high-resolution (0.08 nm or 45 µeV) grating of the spectrometer, while the rest were captured using a lower resolution (0.2 nm or 110 µeV) setup.

tent with droplet-etched QDs in GaAs/AlGaAs, and significantly smaller than strain-based InGaAs/GaAs QDs (typically >25 meV). Individual QD spectra feature narrow and frequently resolution-limited (50 µeV or 0.09 nm) in >90% of QDs analyzed. The reduced inhomogeneous width and sharper homogeneous linewidths confirm smaller variation in the nanohole morphology and suppressed mixing with QW-like states. Moreover, we carried out polarization-resolved spectroscopy measurements and identified that at least 40 out of the 77 QDs analyzed on this sample exhibited measurable fine-structure splitting (FSS) induced oscillation of the neutral exciton's emission frequency. From these measurements, we extract 11 ± 5 µeV FSS with $> 10\%$ of the QDs exhibiting nearly vanishing FSS, indicating highly-symmetric QDs compatible with entangled photon pair generation using biexciton cascade in the telecom band. The FSS measurements are reported in the main text (see Figure 4c,d).

These ensemble measurements of the emission lineshapes and nanohole characterization using AFM substantiate the morphology-optics link: shallow-nanohole QDs favor hybridized, broadened emission with prominent state filling, whereas deeper-nanohole QDs reproducibly exhibit discrete excitonic transitions with narrow linewidths. This broader dataset demonstrates that the single-QD behavior highlighted in the main text is representative of the underlying QD populations.

S6. Spatially-Resolved Emission Spectra

Figure S6a presents the μ-PL spectra collected at two distinct locations on *Sample A*: (1) directly at a single QD site and (2) 5 µm away in a QD-free region in the fluorescence image. The average laser power was held constant at -24 dBm (for reference, the relation $P_{\mathrm{dBm}} = 10 \log_{10} P_{\mathrm{mW}}$ is used to convert power from milliwatts to dBm). Away from the QD, the emission is centered at 1318 nm (Figure S6a), consistent with the exciton binding energy of a 6.3 nm GaSb QW. At the QD site, QW emission is suppressed and exhibits a 6 nm redshift, indicating localized perturbation of the QW optical properties.

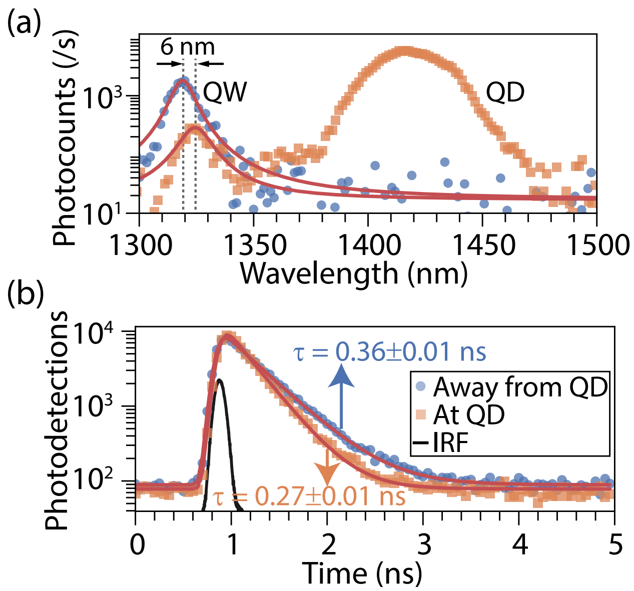

Figure S6: **(a)** μ-PL spectra collected from the QW emission region of *Sample A* when exciting away from a QD (blue circles) and at a QD site (orange squares). The excitation power was held constant at -24 dBm. Lorentzian fits (red curves) reveal a 6 nm redshift at the QD site. **(b)** Time-resolved QW fluorescence, spectrally filtered with a 0.2 nm bandpass centered at the respective peak emission wavelengths, and fitted with single-exponential decays (red curves).

Time-resolved μ-PL measurements of the QW emission were performed at the respective peak wavelengths, selected using a 0.2 nm bandpass filter (Figure S6b). Fitting the data to a single-exponential decay convolved with a Gaussian point spread function (110 ps full-width at half maximum) revealed a 30% faster decay rate at the QD location. This accelerated decay suggests additional nonradiative or charge-transfer pathways from the QW in the vicinity of a QD, consistent with local electronic coupling between these nanostructures. We note, however, that the observed lifetime differences could alternatively arise from the modulation of the QW thickness in the vicinity of the probed QD site, which remains unresolved within the diffraction-limit ($\approx$ 1.1 µm) of the optical setup.

To further probe thermal effects in this GaSb/AlGaSb QW-QD coupled nanostructure, we measured temperature-dependent μ-PL dynamics of the QD emission (Figure S7). At low temperatures and under weak non-resonant excitation (-24 dBm), the decay is well described by a single exponential. This behavior is consistent with rapid QW to QD transfer

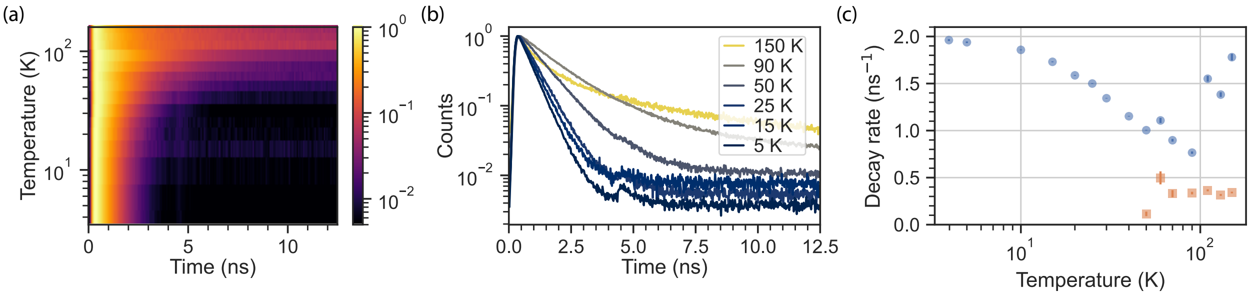

Figure S7: **(a)** Temperature-dependent time-resolved μ-PL under non-resonant excitation (-24 dBm). Each trace is normalized to its maximum at that temperature. **(b)** Representative traces at selected temperatures. **(c)** Fitted decay rates versus temperature (blue: fast and, where applicable, orange: slow components).

and negligible state-filling under weak excitation, yielding a single dominant timescale for the hybridized emitter (see also the weak-power time-resolved PL in Figure 3c, main text). With increasing temperature, the decay rate slows and becomes measurably biexponential by 50 K, which we hypothesize to the onset of thermally-assisted exchange with QW-like/shallow localized states (reservoir-assisted repopulation). A more detailed, quantitative analysis is left for future work and will require temperature-dependent electronic-structure calculations together with temperature- and power-dependent emission analysis at both the QW and QD wavelengths. Above 100 K, additional non-radiative escape channels activate (e.g., thermal escape), accelerating the fast component while a slow tail persists due to continued reservoir coupling. Overall, these trends are consistent with the QW–QD coupled nanostructure picture and complement the time- and wavelength-resolved results presented in the main text (Figure 3).

S7. Four-Level Rate Equation Model of Coupled QD-QW Nanostructures

We employed a four-level rate-equation model to quantitatively analyze exciton recombination dynamics and charge transfer from the QW to the QD. Since we investigate these dynamics under non-resonant excitation, we assume that optical coherences are rapidly lost

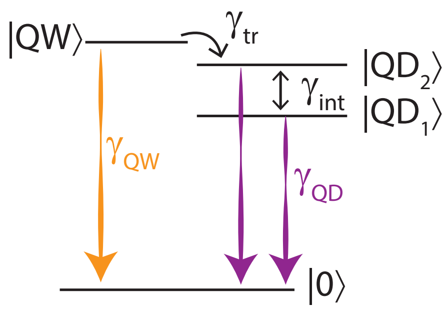

Figure S8: The 4-level system (reproduced from the main text) employed to model the time-resolved photoluminescence of QDs in *Sample A*. Key parameters of this model are the excitation transfer rate from QW into the QD excited state γ_{tr}, QD intersublevel relaxation rate γ_{int}, and radiative decay rates of the quantum well γ_{QW} and quantum dot γ_{QD}.

during the early stages of carrier relaxation and neglect coherence terms between the excitation field, $\Omega(t, P_{\mathrm{exc}})$, and the state populations. Consequently, our analysis is based on incoherent rate equations describing carrier populations, following the energy scheme in Fig. S8. For simplicity, we describe the tunneling and relaxation dynamics using an excitonic picture. While this approximation neglects the more complex electron and hole dynamics originating from the disparity in their effective masses, previous studies showed that it captures the effective dynamics of excitons.[6]

We define the occupation numbers n_i for each energy state i ($i = 0$ for the ground state and $i = \mathrm{QD1}, \mathrm{QD2}$ for the first and second excited states of the QD) and n_{QW} for the QW. Under non-resonant optical excitation with average power P_{exc}, carriers are generated in the $Al_{0.3}Ga_{0.7}AsSb$ barrier material, from which they relax and become predominantly trapped in the QW. The QW is coupled to the excited state of the QD, transferring carriers at a rate γ_{tr}, which modifies the QD occupation numbers. We do not specify the underlying microscopic mechanism of this transfer. The rate equations governing the occupation numbers of these

states are given by:

$$\frac{dn_{\mathrm{QW}}}{dt} = \Omega(t, P_{\mathrm{exc}}) - \gamma_{\mathrm{QW}} n_{\mathrm{QW}} - \gamma_{\mathrm{tr}} n_{\mathrm{QW}} \left(1 - \frac{n_{\mathrm{QD2}}}{2}\right), \tag{1}$$

$$\frac{dn_{\mathrm{QD2}}}{dt} = \gamma_{\mathrm{tr}} n_{\mathrm{QW}} \left(1 - \frac{n_{\mathrm{QD2}}}{2}\right) - (1 - n_0)\gamma_{\mathrm{int}} n_{\mathrm{QD2}} - \left(1 - \frac{n_{\mathrm{QD1}}}{2}\right) \gamma_{\mathrm{int}} n_{\mathrm{QD2}} - \gamma_{\mathrm{QD}} n_{\mathrm{QD2}}, \tag{2}$$

$$\frac{dn_{\mathrm{QD1}}}{dt} = \left(1 - \frac{n_{\mathrm{QD1}}}{2}\right) \gamma_{\mathrm{int}} n_{\mathrm{QD2}} - (1 - n_0)\gamma_{\mathrm{int}} n_{\mathrm{QD1}} - \gamma_{\mathrm{QD}} n_{\mathrm{QD1}}. \tag{3}$$

Here, γ_{QW} and γ_{QD} denote the radiative recombination rates of excitons in the QW and QD, respectively. Non-radiative rates are neglected as they are assumed to be insignificant compared to other dynamics. The intersublevel relaxation rate between QD states is given by γ_{int}. Carrier transfer to the QD is constrained by Pauli blocking, requiring available states for occupation. To account for this, the probability that a state is unoccupied, $(1 - n_i/2)$, modifies the transfer rate. The factor of 2 in the denominator captures the spin degeneracy of the QD exciton states.

When fitting the population dynamics of $|\mathrm{QD}_1\rangle$ and $|\mathrm{QD}_2\rangle$ states to the measured time-resolved photoluminescence data, the model output was convolved with a Gaussian function to account for the timing jitter of the SNSPDs. The full width at half maximum (FWHM) of this Gaussian was $90-130$ ps, depending on the specific detector. This timing resolution was characterized by sending attenuated ultrashort optical pulses to the SNSPDs and measuring their response.

To fit the model to the experimental data, the system of differential equations was solved using SciPy,[7] which numerically integrates the rate equations over time. The fitting process employed SciPy's optimization routines to find the maximum likelihood estimates of the model parameters. All eight datasets were simultaneously fit to the model, resulting in excellent agreement with the experimental data as discussed in the main text. This global fitting approach ensured a consistent set of parameters that accurately captured the dynamics of exciton recombination and charge transfer across different experimental conditions.

S8. Three-level rate equation model of bright-dark QD exciton dynamics

The QD neutral exciton forms from the single-particle states of an electron and a heavy hole, with angular momenta of $\pm 1/2$ and $\pm 3/2$ along the growth axis, respectively. We neglect the contribution from light-holes as quantum confinement lifts the valence band degeneracy leading to heavy-hole states being energetically favorable in as-grown unstrained nanohole-etched QDs.[8] The electron and heavy-hole states combine to create four excitonic states characterized by total angular momenta J of ± 1 or ± 2. Since photons carry an angular momentum of ± 1, only excitons with $J = \pm 1$ can radiatively recombine while conversing the angular momentum. These states are known as bright excitons, whereas the states with $J = \pm 2$, which do not couple to photons, are referred to as dark excitons. The electron-hole exchange interaction lifts the degeneracy of these exciton states, introducing an energy splitting between the bright and dark excitons, with the dark exciton typically having a slightly weaker binding energy by approximately 0.3 meV.[9] Additionally, within the bright (or dark) exciton manifold, fine-structure splitting (FSS) arising from asymmetries in the QD confinement potential can split the states with different in-plane spin projections. In high-symmetry droplet-etched QDs, the in-plane asymmetry is significantly reduced, resulting in a near-zero fine-structure splitting (about 10 µeV) between the bright exciton states.[10,11] However, the bright-dark exciton splitting due to exchange interactions remains finite, ensuring that dark excitons remain lower in energy.

Given this structure, the exciton population dynamics in such QDs can be effectively modeled using the three-level system shown in Fig. S9, where we consider a single bright exciton state and a single dark exciton state.[12] Since we investigate exciton dynamics under non-resonant excitation, we assume that optical coherences are rapidly lost, allowing us to neglect coherence terms between the excitation field and the state populations. Thus, the system can be described using incoherent rate equations for the exciton populations. We

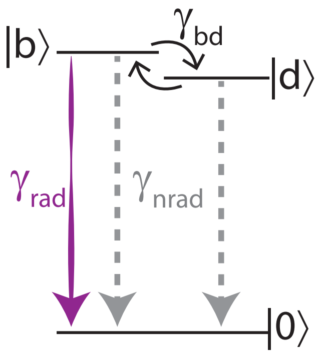

Figure S9: The 3-level system (reproduced from the main text) employed to model the observed time-resolved photoluminescence from a single QD from *Sample B*. Key parameters of this model are the bright to dark (or dark to bright) exciton spin-flip rate γ_{bd}, radiative decay rate γ_{QD}, and the non-radiative decay rate γ_{nrad}.

define the state occupation numbers n_i, where $i = 0$ corresponds to the ground state, and $i = b, d$ correspond to the bright and dark exciton states, respectively. Under non-resonant excitation with an average power P_{exc}, the excitation field $\Omega(t, P_{\text{exc}})$ populates the bright and dark states equally, as carrier capture is dominated by incoherent charge tunneling from the barrier material. The rate equations governing the population dynamics are:[13]

$$\frac{dn_b}{dt} = \Omega(t, P_{\text{exc}}) - (\gamma_{\text{rad}} + \gamma_{\text{nrad}} + \gamma_{\text{bd}})\, n_b + \gamma_{\text{bd}} n_d, \tag{4}$$

$$\frac{dn_d}{dt} = \Omega(t, P_{\text{exc}}) - (\gamma_{\text{nrad}} + \gamma_{\text{bd}})\, n_d + \gamma_{\text{bd}} n_b. \tag{5}$$

Here, γ_{bd} is the spin-flip rate between the bright $|b\rangle$ and dark $|d\rangle$ exciton states, γ_{rad} is the radiative decay rate of the bright exciton, and γ_{nrad} represents the non-radiative decay rate, which we assume to be the same for both bright and dark states. Spin flips in semiconductor materials are governed by various mechanisms, including heavy- and light-hole mixing (Elliott-Yafet mechanism), spin-orbit coupling combined with phonon scattering, carrier scattering, and hyperfine interactions with lattice nuclear spins.[14] While strong quantum confinement suppresses Elliott-Yafet contributions due to the non-degenerate light and heavy hole bands, the remaining mechanisms likely contribute in descending order of importance.

Solving the system of coupled rate equations yields a biexponential decay for the time-resolved spontaneous emission from the bright exciton state:

$$I(t) = A_{\mathrm{b}} e^{-\gamma_{\mathrm{b}} t} + A_{\mathrm{d}} e^{-\gamma_{\mathrm{d}} t}, \tag{6}$$

where the decay rates for the bright and dark excitons are:

$$\gamma_{\mathrm{b}} = \gamma_{\mathrm{rad}} + \gamma_{\mathrm{nrad}} + \gamma_{\mathrm{bd}}, \tag{7}$$

$$\gamma_{\mathrm{d}} = \gamma_{\mathrm{nrad}} + \gamma_{\mathrm{bd}}. \tag{8}$$

The amplitudes of the bright and dark components in the biexponential decay are given by:

$$A_b = \gamma_{\mathrm{rad}} n_b(0) - \gamma_{\mathrm{bd}} n_d(0), \tag{9}$$

$$A_d = \gamma_{\mathrm{bd}} n_d(0), \tag{10}$$

where $n_b(0)$ and $n_d(0)$ are the initial populations of the bright and dark states immediately after excitation by a short pulse $\Omega(t, P_{\mathrm{exc}})$, much shorter than $1/\gamma_{\mathrm{rad}}$. The relative amplitudes of the fast and slow decay components are directly influenced by the spin-flip rate γ_{bd}, making this model a powerful tool for investigating dark exciton dynamics and spin relaxation processes in quantum dots. By fitting the experimental time-resolved photoluminescence data to this model, one can extract key parameters such as the spin-flip rate, radiative and non-radiative decay rates, and their dependence on the QD environment. Notably, this technique has been widely applied to probe spin relaxation dynamics in nanophotonic structures, where dark exciton lifetimes are significantly extended due to suppressed radiative recombination.[15]

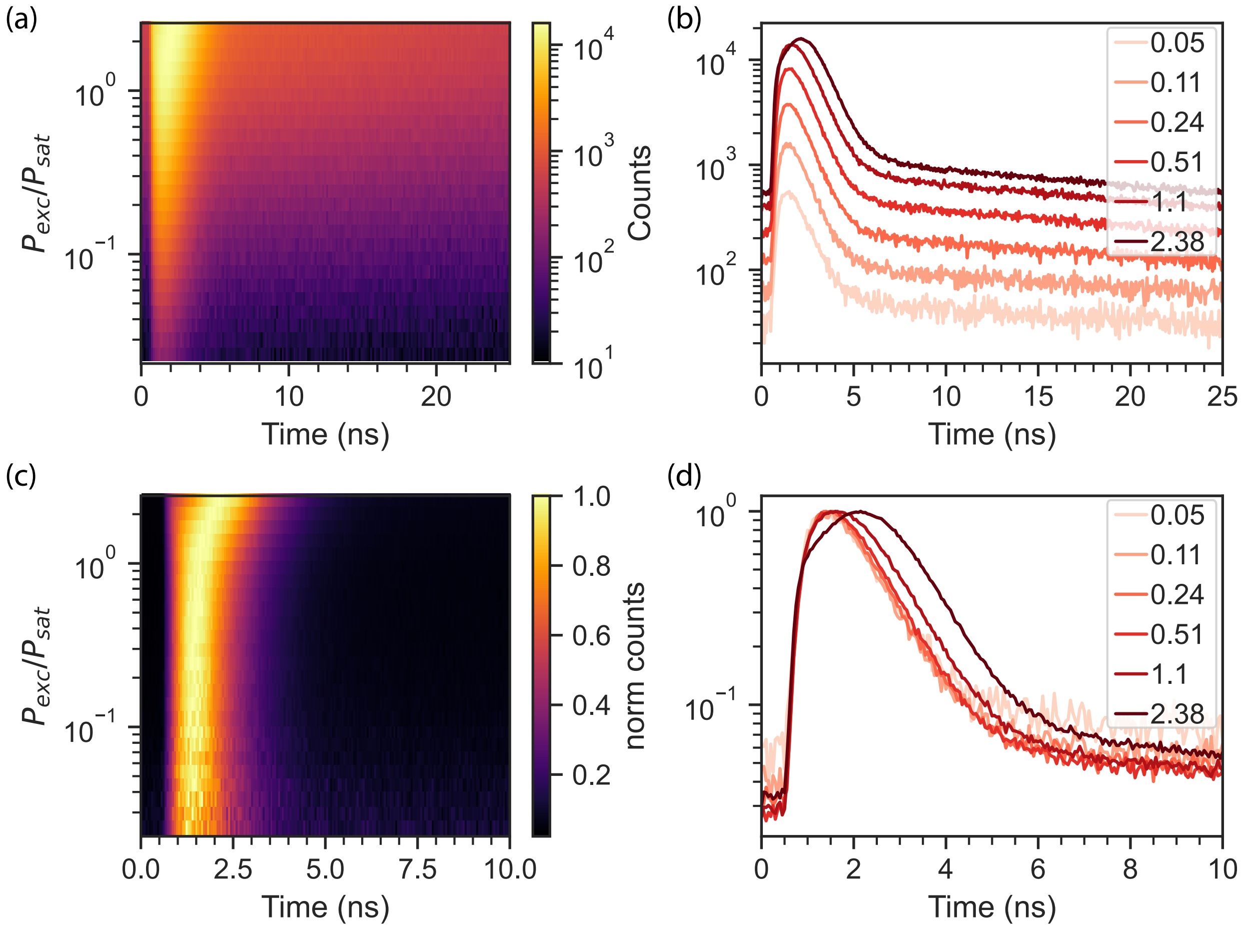

Figure S10: Power-dependent time-resolved exciton emission. **(a)** Two-dimensional map of the exciton photoluminescence as a function of excitation power P_{exc}, normalized to the saturation power P_{sat}, shown on a logarithmic color scale. **(b)** Representative traces at selected excitation powers (legend indicates $P_{\mathrm{exc}}/P_{\mathrm{sat}}$), highlighting the biexponential dynamics up to P_{sat} and the onset of broadening only above saturation. **(c)** Normalized colormap of the same data, restricted to the 0-10 ns window to emphasize temporal dynamics. **(d)** Corresponding normalized traces illustrating the power-dependent modification of the emission profile.

S9. Power-dependent time-resolved μ-PL on *Sample B*

Figure S10 extends the neutral-exciton TRPL data discussed in Figures 4–6 of the main text. Figure S10(a) shows the full dataset on a logarithmic color scale to capture the wide dynamic range of emission intensities. Selected traces in panel (b) highlight that the decay remains well-described by a biexponential up to $P_{\rm sat}$, while higher excitation produces clear broadening. To better isolate temporal dynamics, each trace was normalized to its maximum in panels (c,d). The normalized map in (c), limited to the first 10 ns, emphasizes the excitation-induced temporal shift, while the traces in (d) confirm that additional features appear only under strongly above-saturation pumping. These results support the main-text conclusion that intrinsic exciton dynamics remain clean until perturbed by high-power excitation. Strong non-resonant excitation creates a high density of charge carriers in the barrier layer, which can rapidly transfer to the QD, resulting in state-filling-like behavior.

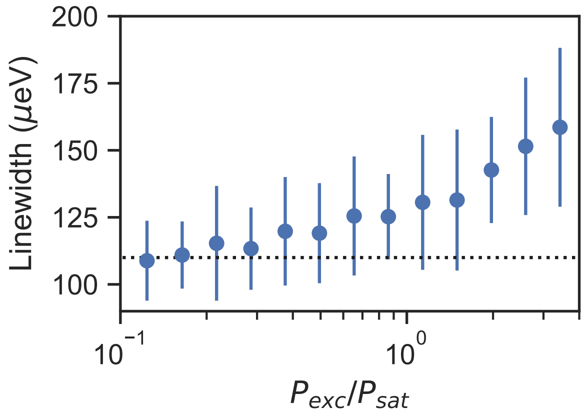

Figure S11: Power-dependent linewidth of the exciton peak. The linewidth (Gaussian FWHM) is plotted as a function of the normalized exciton power. The dashed line denotes the resolution limit of the spectrometer.

To complement the time-resolved measurements, Figure S11 presents the power-dependent evolution of the exciton emission linewidth extracted from Gaussian fits to the μ-PL spectra. At low excitation powers, the linewidth remains close to the instrumental resolution limit (dashed line), indicating negligible additional broadening. Upon approaching and exceed-

ing the saturation power P_sat, a clear increase in linewidth is observed. This behavior is consistent with excitation-induced dephasing mechanisms arising from enhanced carrier populations in the nearby wetting layer or barrier, which can interact with the confined exciton state. The gradual broadening thus supports the interpretation that non-radiative interactions and state-filling effects become relevant only under high-power excitation conditions.

References

1. Pimpinelli, A.; Einstein, T. L. *Phys. Rev. Lett.* **2007**, *99*, 226102.

2. González, D. L.; Pimpinelli, A.; Einstein, T. L. *Phys. Rev. E* **2011**, *84*, 011601.

3. Einstein, T. L.; Pimpinelli, A.; Luis González, D. *Journal of Crystal Growth* **2014**, *401*, 67–71.

4. Löbl, M. C.; Zhai, L.; Jahn, J.-P.; Ritzmann, J.; Huo, Y.; Wieck, A. D.; Schmidt, O. G.; Ludwig, A.; Rastelli, A.; Warburton, R. J. *Phys. Rev. B* **2019**, *100*, 155402.

5. Leguay, L.; Chellu, A.; Hilska, J.; Luna, E.; Schliwa, A.; Guina, M.; Hakkarainen, T. *Mater. Quantum Technol.* **2024**, *4*, 015401.

6. Mazur, Y. I.; Dorogan, V. G.; Marega, E.; Zhuchenko, Z. Y.; Ware, M. E.; Benamara, M.; Tarasov, G. G.; Vasa, P.; Lienau, C.; Salamo, G. J. *J. Appl. Phys.* **2010**, *108*, 074316.

7. Virtanen, P.; Gommers, R.; Oliphant, T. E.; Haberland, M.; Reddy, T.; Cournapeau, D.; Burovski, E.; Peterson, P.; Weckesser, W.; Bright, J.; van der Walt, S. J.; Brett, M.; Wilson, J.; Millman, K. J.; Mayorov, N.; Nelson, A. R. J.; Jones, E.; Kern, R.; Larson, E.; Carey, C. J. *et al. Nat. Methods* **2020**, *17*, 261–272.

8. Huo, Y. H.; Witek, B. J.; Kumar, S.; Cardenas, J. R.; Zhang, J. X.; Akopian, N.;

Singh, R.; Zallo, E.; Grifone, R.; Kriegner, D.; Trotta, R.; Ding, F.; Stangl, J.; Zwiller, V.; Bester, G.; Rastelli, A.; Schmidt, O. G. *Nat. Phys.* **2013**, *10*, 46–51.

9. Bayer, M.; Ortner, G.; Stern, O.; Kuther, A.; Gorbunov, A. A.; Forchel, A.; Hawrylak, P.; Fafard, S.; Hinzer, K.; Reinecke, T. L.; Walck, S. N.; Reithmaier, J. P.; Klopf, F.; Schäfer, F. *Phys. Rev. B* **2002**, *65*, 195315.

10. Huo, Y. H.; Rastelli, A.; Schmidt, O. G. *Appl. Phys. Lett.* **2013**, *102*, 152105.

11. Michl, J.; Peniakov, G.; Pfenning, A.; Hilska, J.; Chellu, A.; Bader, A.; Guina, M.; Höfling, S.; Hakkarainen, T.; Huber-Loyola, T. *Adv. Quantum Technol.* **2023**, *6*, 2300180.

12. Johansen, J.; Julsgaard, B.; Stobbe, S.; Hvam, J. M.; Lodahl, P. *Phys. Rev. B* **2010**, *81*, 081304.

13. Johansen, J. Decay Dynamics of Quantum Dots in Nanophotonic Structures. Ph.D. thesis, Technical University of Denmark, 2008.

14. Liao, Y.-H.; Climente, J. I.; Cheng, S.-J. *Phys. Rev. B* **2011**, *83*, 165317.

15. Wang, Q.; Stobbe, S.; Lodahl, P. *Phys. Rev. Lett.* **2011**, *107*, 167404.
